# Reporting Guidelines for the Early-Phase Clinical Evaluation of Applications Using Extended Reality: RATE-XR Qualitative Study Guideline

**DOI:** 10.2196/56790

**Published:** 2024-11-29

**Authors:** Johan H Vlake, Denzel L Q Drop, Jasper Van Bommel, Giuseppe Riva, Brenda K Wiederhold, Pietro Cipresso, Albert S Rizzo, Barbara O Rothbaum, Cristina Botella, Lotty Hooft, Oscar J Bienvenu, Christian Jung, Bart Geerts, Evert-Jan Wils, Diederik Gommers, Michel E van Genderen, RATE-XR Expert Group

**Affiliations:** 1 Department of Intensive Care Erasmus Medical Center Rotterdam Netherlands; 2 Department of Intensive Care Franciscus Gasthuis & Vlietland Rotterdam Netherlands; 3 Applied Technology for Neuro-Psychology Lab IRCCS Istituto Auxologico Italiano Milan Italy; 4 Department of Psychology Catholic University of the Sacred Heart Milan Italy; 5 Virtual Reality Medical Center San Diego, CA United States; 6 Department of Psychology University of Turin Turin Italy; 7 Medical Virtual Reality Lab University of Southern California Institute for Creative Technologies Los Angeles, CA United States; 8 Department of Psychiatry and Behavioral Sciences Emory University School of Medicine Atlanta, GA United States; 9 Department of Basic Psychology, Clinic, and Psychobiology University Jaume I Castellón Spain; 10 CIBER de Fisiopatología de la Obesidad y Nutrición (CIBEROBN) Instituto Salud Carlos III Madrid Spain; 11 Department of Epidemiology, Julius Center for Health Sciences and Primary Care University Medical Center Utrecht Utrecht Netherlands; 12 Department of Psychiatry and Behavioral Sciences Johns Hopkins University School of Medicine Baltimore, MD United States; 13 Department of Cardiology, Pulmonology, and Vascular Medicine Medical Faculty, University Hospital Düsseldorf Heinrich-Heine-University Düsseldorf Düsseldorf Germany; 14 Cardiovascular Research Institute Düsseldorf (CARID) Medical Faculty, University Hospital of Düsseldorf Heinrich-Heine University Düsseldorf Düsseldorf Germany; 15 R&D BV Healthplus.ai Amsterdam Netherlands; 16 See Acknowledgments

**Keywords:** extended reality, XR, virtual reality, augmented reality, mixed reality, reporting guideline, Delphi process, consensus, computer-generated simulation, simulation, virtual world, simulation experience, clinical evaluation

## Abstract

**Background:**

Extended reality (XR), encompassing technologies such as virtual reality, augmented reality, and mixed reality, has rapidly gained prominence in health care. However, existing XR research often lacks rigor, proper controls, and standardization.

**Objective:**

To address this and to enhance the transparency and quality of reporting in early-phase clinical evaluations of XR applications, we present the “Reporting for the early-phase clinical evaluation of applications using extended reality” (RATE-XR) guideline.

**Methods:**

We conducted a 2-round modified Delphi process involving experts from diverse stakeholder categories, and the RATE-XR is therefore the result of a consensus-based, multistakeholder effort.

**Results:**

The guideline comprises 17 XR-specific (composed of 18 subitems) and 14 generic reporting items, each with a complementary Explanation & Elaboration section.

**Conclusions:**

The items encompass critical aspects of XR research, from clinical utility and safety to human factors and ethics. By offering a comprehensive checklist for reporting, the RATE-XR guideline facilitates robust assessment and replication of early-stage clinical XR studies. It underscores the need for transparency, patient-centeredness, and balanced evaluation of the applications of XR in health care. By providing an actionable checklist of minimal reporting items, this guideline will facilitate the responsible development and integration of XR technologies into health care and related fields.

## Introduction

Extended reality (XR) encompasses various forms of computer-generated reality, including augmented reality (AR), mixed reality (MR), and virtual reality (VR). XR, mainly in the form of VR, has rapidly emerged in health care, particularly in fields such as mental health, intensive care medicine, surgery, pain management, and rehabilitation [1-4]. Much like other transformative technologies as artificial intelligence (AI) algorithms, the field of XR has witnessed an exponential surge in research and applications: from 1992 to 2005, merely up to 100 publications were recorded yearly, but this number has steadily increased, with over 1000 publications annually since 2018 [2]. Notably, the US Food and Drug Administration has been approving a growing number of XR-based devices, underscoring its escalating clinical significance [5].

Despite this expanding landscape of XR research in health care, most studies primarily focus on treatment effects, tend to be small and heterogeneous, and often lack proper control conditions [6]. Consequently, comparing XR studies is challenging, as scientific rigor is often lacking and they pose several unique implementation and technological challenges in health care [7-9]. To overcome these challenges and to promote optimal reporting of XR-based interventions' clinical utility, a more structured approach is essential. This approach should encompass technological, methodological, and safety aspects to support a more objective understanding of the validity and generalizability of findings [10]. The challenges of early-stage clinical evaluation of applications using XR (Textbox 1) share similarities to those of other innovative technologies and interventions such as developing and implementing surgical innovations or AI models [11-14].

**Figure 1:**
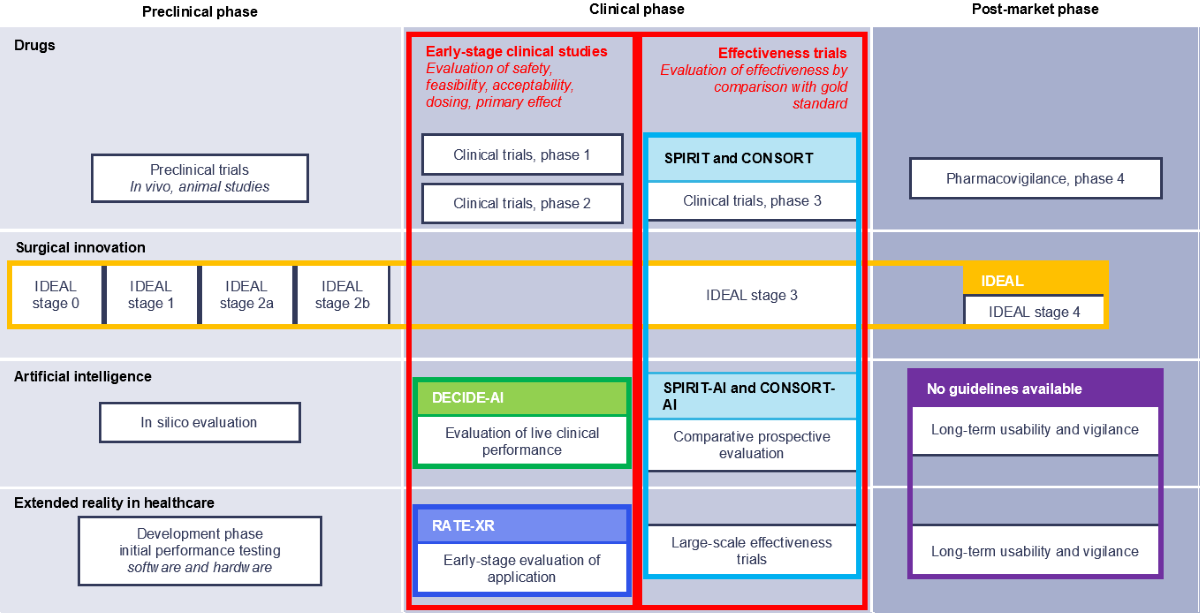
Comparison of development pathways for drug therapies, surgical innovation, artificial intelligence, and extended reality in health care. The colored lines represent reporting guidelines, some of which are study design–specific (SPIRIT or CONSORT and SPIRIT or CONSORT-AI); others are stage-specific (IDEAL and RATE-XR). Depending on the context, more than one study design can be appropriate for each stage. CONSORT: Consolidated Standards of Reporting Trials; RATE-XR: reporting for the early-phase clinical evaluation of applications using extended reality; SPIRIT: Standard Protocol Items: Recommendations for Interventional Trials.

Early-stage clinical evaluation of XR systems plays a pivotal role in bridging the gap between preclinical technological development and large-scale effectiveness trials. Existing guidelines such as the VR core model stage 2/3 recommendations, SPIRIT (Standard Protocol Items: Recommendations for Interventional Trials) and CONSORT (Consolidated Standards of Reporting Trials) statements, including their AI extensions, and the IDEAL guidelines offer valuable insights into the design and reporting of clinical trials (Figure 1) [12,13,15,16]. However, they generally focus on later stages of clinical research or do not adequately address the specific challenges of developing and evaluating XR technologies in early clinical settings. These include the rapid evolution of XR hardware and software and the specific safety, usability, and ethical considerations that arise in these contexts. To address these gaps, the reporting for the early-phase clinical evaluation of applications using extended reality (RATE-XR) guideline specifically tailors its recommendations to support transparent reporting and effective evaluation of XR applications from the developmental phase through to early clinical trials, ensuring that these innovations can be safely and effectively integrated into health care practice.

**The challenges of early-phase clinical evaluation of applications using extended reality (XR). the clinical evaluation of applications using XR presents several challenges, all of which will likely be encountered at early stages. This textbox represents several of these challenges.**

- Allow for a continuous changing nature of the software applications using XR and its hardware (due to early prototyping and version updates)
- Account for technical errors negatively impacting the reliability and consistency of clinical evaluations
- Evaluate the generalizability of findings across sites and populations
- Deal with ethical considerations in this early stage of research
- Deal with a variety of clinical trial endpoints for applications due to the wide range of intended uses
- Account for user variability and consequently the arising bias as users may not be trained adequately or may not be familiar with XR technology
- Incorporate XR applications into the standard workflow
- Absence of established methodologies and frameworks
- Having sufficient image quality of XR devices for users, due to their diversity and constantly evolving technological characteristics
- Create a usability profile applicable to various working environments

Early-stage clinical evaluation of XR interventions must prioritize clinical utility, safety, and human factors challenges in real-life clinical settings. Factors such as cybersickness, customizability and duration of the XR content, treatment frequency, and immersiveness contribute to safety and feasibility considerations and must be addressed transparently. A one-size-fits-all model is often not feasible, and neglecting safety profiles and rushing into large-scale trials can jeopardize patient well-being, which is ethically unacceptable. In terms of ethics, patient-centeredness and commercial interests must be addressed in the early stages of XR development. Currently, studies too often prioritize assessing commercial products but fail to address essential clinical research elements or tailoring content for medical applications, while understanding the interaction between XR technology and human factors is essential [17-19]. The clinical context should be the starting point in the development of medical XR systems, involving patients and health care providers as primary stakeholders early in the process to design systems that optimally address their needs, beyond placing an initial focus on commercial product evaluation. Moreover, variations in hardware, software, and content selection are complex to assess and often underreported [20].

To address these challenges and with the aim of improving the consistency, safety, knowledge generation, and applicability of XR research in the health care domain, we undertook a robust 2-phase modified Delphi process. This collaborative effort engaged strong and diverse stakeholder engagement and resulted in the development of the RATE-XR guideline. Here, we present the development process, key recommendations, and their implications for the XR health care field.

## Methods

### RATE-XR Guideline Development

The RATE-XR guideline was developed through an international expert consensus process adhering to the EQUATOR Network’s recommendations for guideline development [21].

### Establishment of the Steering Committee

To guide the development of the RATE-XR guideline, a Steering Committee was assembled, detailed in Table S1 in Multimedia Appendix 1. The committee was selected by the project initiators—JHV, JvB, and MEvG—to ensure a diverse representation of expertise within the XR and research domains. Members of our study group, including CJ, DLQD, DG, EJW, and OJB, were included for their direct contributions to this project. Additionally, we engaged 5 of the most influential authors in XR research as cited in a recent JMIR article—GR, BKW, PC, ASR, and CB—to incorporate a broad range of perspectives and expertise [2]. We also invited BOR, a pioneer in clinical VR, and BJB, known for his work in VR trial design and implementation. Finally, to incorporate expertise in methodology and guideline development, LH and BG, both experienced in developing previous reporting guidelines, were included [13]. We conducted a modified Delphi process based on previous guideline development consisting of 2 rounds of feedback from participating experts followed by virtual consensus meetings and qualitative evaluation by an independent evaluation committee [13,22,23].

### Ethical Considerations

The project was approved by the Medical Ethics Committee of the Erasmus Medical Centre, Rotterdam (approval 2022-0623) and registered with the EQUATOR (Enhancing the Quality and Transparency of Health Research) network. Informed consent was obtained from all members of the Steering Committee, all participants of the Delphi rounds, and all members of the evaluation committee.

### Generation of the Initial Item List

An initial list of 61 candidate items (with subitems) was composed by 2 authors (JV and MEvG) and was based on (1) scientific reports on trials examining XR-based studies in health care [24-27], (2) recently published innovative technology guidelines [13,28], (3) methodological and evaluative challenges concerning the application of XR in health care [14,16], (4) a Cochrane Systematic Review on the clinical use of XR [29], and (5) institutional documents [30-32]. Hereafter, the candidate item list was commented on by the Steering Group members (Steering Group Round).

### Expert Recruitment

Experts were recruited using five distinct approaches: (1) invitations to experts that were endorsed by the Steering Group members, (2) invitations to authors of publications identified through the preliminary literature search, (3) a call for contributions published within a medical journal [33], (4) consideration of professionals proactively reaching out to the Steering Group, and (5) invitations to experts recommended by the Delphi participants (snowballing). Prior to the initiation of recruitment, 17 target stakeholder groups were defined: clinicians, engineers or computer scientists, methodologists, statisticians, implementation specialists, entrepreneurs, epidemiologists, journal editors, allied health professionals, policy makers or official institutional staff, administrators or hospital management, researchers, ethicists, private sector representatives, patient representatives, funders, and psychologists or psychiatrists.

### The Delphi Process

The Delphi process consisted of 2 rounds, and the Delphi surveys were designed and distributed using the Castor Electronic Data Capture web application (Castor EDC). The first round encompassed 2 parts. In the first part, participants answered 6 open-ended inquiries that address facets considered essential to be reported on during early-phase clinical evaluation. In the second part, Delphi participants were tasked with rating, from 1 to 9, the significance of items in the initial list. Ratings of 1 to 3 indicated insignificance, 4 to 6 denoted importance without being pivotal, and 7 to 9 implied that items were both important and critical. In addition to rating the items, participants were prompted to offer commentary on items and propose new additions. Thematic analysis of the open-ended questions was independently conducted by 2 Steering Group members (JV and MEvG), with any disagreements being resolved through consensus. Identified themes were used to determine whether any important themes were missing in the item list, along with newly proposed items to complement the item list. Hereafter, a summary score, including the median, 25th percentile, 75th percentile, mean, SD, proportion of participants scoring the item above 7 or below 3, and stakeholder groups with a median of ≤2 or ≥2 points from the overall median were calculated for each item. Prespecified inclusion cutoffs were determined as an item scoring a mean ≥7 and exclusion as an item scoring a mean ≤3. Based on these results, a revised item list for the second Delphi round was generated.

In the second Delphi round, participants were presented with the outcomes of the first round, along with the revised item list. Participants were tasked with reevaluating all items in a manner akin to the first Delphi round and were invited to comment on content and wording. Both Delphi round surveys and outcomes are accessible through the Open Science Framework (OSF) [34]. All analyses were performed using R for Statistics (R Foundation for Statistical Computing).

### Consensus Meeting

Virtual consensus meetings were held on 3 separate occasions between June 12 and 15, 2023, with the aim of finalizing content and refining the phrasing of items within the RATE-XR reporting guideline. To ensure a balanced representation of key stakeholders throughout the XR field and geographic diversity, we engaged 18 experts with diverse expertise and backgrounds (Tables S2-S4 in Multimedia Appendix 1). Throughout the consensus meetings, all items from the second Delphi round were subject to discussion and anonymous voting, facilitated by the Mentimeter platform [35]. The voting process was overseen by a chairman and observed by a designated observer. For an item to be ultimately included in the definitive guideline, a predefined threshold of 80% agreement among Consensus Group members was necessary, excluding abstentions and blank votes.

### Qualitative Evaluation

After finalizing both the guideline and the Explanation & Elaboration note, a qualitative evaluation was conducted by a panel of 14 experts (Note 1 in Multimedia Appendix 1) possessing significant experience in implementing or peer-reviewing literature relevant to applications using XR. None of the experts involved in the qualitative evaluation were affiliated with the Consensus Group. Their input focused on evaluating the clarity and usability of each XR-specific item using a custom form, which is available on the OSF platform [34]. In total, 3 reviewers (JV, DD, and MEvG) independently reviewed the provided comments to assess the necessity for revisions in the wording of items or their corresponding Explanation & Elaboration sections. The review process was structured to ensure comprehensive coverage and unbiased analysis of the feedback. Disagreements between reviewers were resolved through consensus, ensuring a balanced interpretation of the qualitative data. This methodological rigor enhances the reliability of the modifications made to the RATE-XR guidelines based on stakeholder feedback. To enhance comprehension of key concepts within the guideline, a glossary of terms (Table 1) was composed. All Consensus Group members approved the modifications, the final guideline, and the complementary Explanation & Elaboration note.

**Table 1:** Glossary of terms^a^.

Terms	Explanations
Application	The software, program, intervention, or modality using an XR device or hardware.
Artificial intelligence algorithm	”Science of developing computer systems which can perform tasks normally requiring human intelligence” based on a mathematical model responsible for learning from data and producing an output [36].
Augmented reality	A technology that overlays digital information onto the real-world environment, viewed through an augmented reality headset or glasses in order to enhance the user’s perception of reality. Augmented reality is part of the extended reality (XR) technologies.
Bias	Systematic difference in treatment of certain objects, people, or groups in comparison to others [37].
Clinical utility	The practical value and usefulness of the application using XR.
Commercial name of application	Trademarked or branded name under which the specific software application or hardware device is sold.
Commercial product	An item originally manufactured for sale, lease, or license to the general public.
Cybersickness	A form of motion sickness or discomfort experienced by individuals while using XR devices.
Early-phase studies	Studies in the initial stages of investigation where applications using XR devices are tested and evaluated, focusing on safety, dosage, feasibility, and potential efficacy of the intervention involving a relatively small number of participants.
XR	An umbrella term to encompass the spectrum of immersive technologies consisting of virtual reality, augmented reality, and mixed reality.
Flow diagram	A visual representation using symbols and arrows to illustrate the sequential steps, processes, or decisions within the workflow or procedure.
Hardware	The physical components and equipment that make up the XR system.
Human factors	Also called ergonomics. “The scientific discipline concerned with the understanding of interactions among humans and other elements of a system, and the profession that applies theory, principles, data, and methods to design in order to optimize human well-being and overall system performance” (International Ergonomics Association).
Immersiveness	The degree to which the experience captivates and engages the user, involving a deep sense of presence and absorption within a simulated environment.
Immersive virtual reality	A computer-generated, 3D artificial environment using a head-mounted display and therefore completely surrounding the user’s senses. This way the user is brought from the real world into the artificial, virtual world.
Mixed reality	A technology combining elements of both virtual reality and augmented reality. It integrates digital content and virtual objects into the real-world environment, allowing users to interact with and manipulate these virtual elements as if they were part of their physical surroundings.
Performance	How well the application, device, system, or technology functions and executes its intended task or functionalities.
Preclinical	Pertaining to the phase of research prior to clinical trials targeting actual patients.
Prespecified outcomes	Specific defined results, goals, or expectations that are determined and established in advance, prior to performing the study.
Real clinical setting	Pertaining to the observation and treatment of actual patients, instead of preclinical users or simulated scenarios.
Software	The applications, programs, and digital content are specifically designed to interact with the XR system.
Usability	“Extend to which a product can be used by specified users to achieve specified goals with effectiveness, efficiency, and satisfaction in a specified context of use” [38].

^a^The definitions provided apply to the specific context of RATE-XR and the use of the terms in the guideline. They are not necessarily generally accepted definitions and may not always be entirely suitable for other research domains.

## Results

### Initial Item List

Based on the yielded 97 comments and 22 proposals for new items, 18 items were appended and 29 were subjected to reorganization (through merging or splitting, resulting in 16 items). Additionally, wording was amended, and items were categorized into XR-specific and general items. The final initial item list resulted in 71 Delphi items, subdivided into 41 XR-specific and 30 general reporting items, 6 categories, and 22 subcategories, and was approved on by all Steering Group members (see digital file in the OSF platform) [34].

### Delphi Rounds

A total of 124 individuals expressed their interest and completed the participation form for the first Delphi round, of whom 22 were unqualified due to lack of XR-related experience. Among the 102 experts who received the first Delphi questionnaire, 93 (91%) completed the questionnaire. The participants included 13 Steering Group members, 38 identified from Steering Group recommendations, 13 from proactive contacts or correspondence, and 29 through snowballing. In total, 112 experts were invited to participate in the second Delphi round, of which 96 (86%) responded. In total, 82 of these experts also participated in the first Delphi round (continuity rate: 88%). Collectively, the participating experts represented 14 countries, and all stakeholders were represented (Supplementary Note 1 and Tables S5-S8 in Multimedia Appendix 1).

The first Delphi round yielded over 17,300 words of unstructured text to the open-ended inquiries, along with 6603 item scores, 256 item comments, and 97 newly proposed items. Thematic analysis identified 146 themes, of which 88 were covered in existing items, 22 were integrated into or added to the provisory Explanation & Elaboration note, 28 were used to amend existing items, 2 were selected as new items, and 6 were dropped as they were determined to be outside of the reporting guideline scope. Eventually, 5 items remained unchanged, 27 items were amended or rephrased, 36 items were merged or split into 14 items, 3 items were dropped, and 5 items were added (Figures S1 and S2 in Multimedia Appendix 1). The 3 items that were dropped were related to production costs of the XR module and were dropped due to low consensus in the scoring exercise and congruent comments that these items were out of scope. The revised item list eventually comprised 51 Delphi items in 45 reporting items, subdivided into 22 XR-specific and 23 general reporting items. The second Delphi round yielded 4896 item scores and 372 comments.

### Consensus Meeting

In total, 32 items received endorsement for integration into the RATE-XR guideline during the consensus meetings—17 items specific to XR and 14 encompassing general reporting. A summary of the Consensus Meetings votes is presented in Table S9 in Multimedia Appendix 1.

### Qualitative Evaluation

A total of 95 comments were provided. Subsequently, wording of 7 items was refined in the checklist, and of 9 items, there were modifications in their corresponding Explanation & Elaboration section in Multimedia Appendix 1. The evolutionary trajectory of the item list is presented in Figures S1 and S2 in Multimedia Appendix 1.

### Final Reporting Item Checklist

Table 2 presents the RATE-XR checklist and consists of 17 XR-specific reporting items (composed of 18 subitems) and 14 generic reporting items, selected by the Consensus Group.

**Table 2:** RATE-XR (reporting for the early-phase clinical evaluation of applications using extended reality) checklist.

Theme			Item number^a^		Recommendation
***Title and abstract***					
	Title	1		Identify the study as an early clinical evaluation, or a similar term, of an application using XR^b^, or a more specific term, in the title, including its intended aim.	
	Abstract	I		Provide a (structured) summary of the study. Consider including the following: A concise description of the clinical problem or knowledge gap and the rationale for using an application using XR A concise description of the study methods, including a short description of the application including its name, study population, study setting, main outcomes, and assessment methods. A concise description of the results, including safety and harm outcomes A short conclusion If applicable, details about the registration of the study in a publicly available database.	
***Introduction***					
	Clinical problem and existing evidence	2		Introduce the clinical problem for which the application using XR was used, including its relevance and a description of (the efficacy of) evidence-based or commonly used interventions or the treatment as usual, which is intended to be replaced by the application using XR.	
	Introduction of the application	3		Introduce the application using XR, including the following: Hypotheses for the potential effect; how the application is expected to contribute to the clinical problem. If available, a concise description of, or a reference to, previous research on the same (or a similar) application.	
	Objectives	II		Specify the study objectives or hypotheses.	
***Methods and analysis***					
	Trial design and reporting	III		Provide a reference to ethical approval and, if available, to any (published) study protocol and registration of the study in a publicly available repository.	
	Trial design and reporting	IV		Describe, and mention the rationale for, the study design. For clarification, it is recommended to use a flow diagram.	
	Participants and setting	4		Describe the setting and locations, including country, where data were collected and processed, and where the application using XR was applied and evaluated.	
	Participants and setting	5a		Describe how participants were selected and recruited and provide eligibility criteria.	
	Participants nad setting	5b		Describe who will be applying the application and whether they were trained.	
	Intervention and procedures	6		Provide a description of the application, including its content, hardware, protocol, and set-up, or provide a reference to previous publications where this information is described. Consider supplementing the description with an image, figure, or film.	
	Intervention and procedures	7		Describe, or provide a reference to, the development process of the application.	
	Intervention and procedures	8		Describe the participant timeline in sufficient detail to allow replication, including all procedures, co-interventions (if applicable), and (follow-up) assessments.	
	Intervention and procedures	V		Describe and give a rationale for the control conditions or provide a rationale for not using one.	
	Outcomes	VI		Describe all prespecified primary and secondary outcomes, including how and when assessed.	
	Outcomes	9		Describe how safety and harm outcomes were assessed. Describe which, and how, other XR-specific outcomes were assessed, such as performance, usability, presence, perspectives, and acceptability.	
	Sample size	VII		Provide a justification for the sample size.	
	Analysis	VIII		Provide a detailed description of how primary and secondary outcomes were analyzed, including any prespecified comparisons or stratifications.	
	Protocol alterations	IX		Describe changes to the methods or protocol, including procedures, study outcomes, eligibility criteria, and analysis plan, after study commencement, with reasons, and, if applicable, report whether the study registration was updated.	
***Results***					
	Participant flow and recruitment	X		Describe the time frame of recruitment and follow-up and the participant flow, including the number of patients screened and included, receiving the intervention, and being included in each analysis. Report if, and why, the study was prematurely terminated. The use of a flow diagram is highly recommended.	
	Baseline data	XI		Describe, or add a table depicting, baseline and treatment-related characteristics. If applicable, describe and specify any concurrent measures.	
	Main results	XII		Report on all prespecified outcomes that are available. Consider using tables, figures, or graphs to illustrate results.	
	XR and human factors	10		Include information about the usage of the application, such as duration, frequency, number of sessions, error rates, and number of sessions requiring interruption or discontinuation, including reasons.	
	XR and human factors	11		If assessed, report on XR-specific outcomes, such as performance, usability, presence, perspectives, and acceptability.	
	Safety and harms	12		Report on safety and harms, including unintended effects, both during and after using the application.	
***Discussion and conclusion***					
	Generalizability and impact	13		Discuss (potential) impact of study findings and generalizability, including barriers for the use and implementation of the application.	
	Safety and harms	14		Discuss safety and instances of harm, including their possible effects on study findings, implications for future use of the applications, and whether they can be prevented or mitigated.	
	Ethics	15		Describe ethical considerations, including benefits and risks, for the current and future use of the application.	
	Strengths and limitations	XIII		Discuss study strengths and limitations, including sources of potential bias.	
	Conclusion	16		Provide a conclusion that accurately interprets study findings, including future perspectives.	
***Statements***					
	Funding and conflicts of interest	XIV		Disclose any potential conflict of interest, real or apparent, including the funding sources and their roles in the design, conduct, analysis, and report of the study, potential roles of commercial companies, and personal conflicts of interest for each author.	
	Application	17		Indicate whether the application is a commercial product, it is publicly available, it can be accessed, it complies with the medical device regulations, and whether the application was approved for its intended use by a formal regulatory body or if the study is part of the clinical evaluation for future certification.	

^a^AI-specific items are numbered in Arabic numerals; generic items are numbered in Roman numerals.

^b^XR: extended reality.

## Discussion

### Reporting Item Checklist

The RATE-XR guideline serves as a checklist for reporting studies that focus on the early-phase evaluation of clinical applications using immersive technologies, regardless of the chosen study design (Figure 1). Depending on the specific study design selected, authors may also find it contributing to complement their reporting with guidelines tailored to that study type, such as the CONSORT guideline for randomized trials or the STROBE (Strengthening the Reporting of Observational Studies in Epidemiology) guideline for observational studies [39,40]. This provides a helpful source to support researchers and reviewers assess manuscript compliance with the guideline. For a more elaborate understanding of each item's relevance and recommendations on how to report, we added an in-depth Explanation & Elaboration section for every item in Note 2 in Multimedia Appendix 1.

It is important to recognize that reporting guidelines, including the RATE-XR guideline, offer a framework of reporting recommendations, yet they may not comprehensively cover every aspect relevant to a particular study or guide the conduct of research. While not exhaustive, familiarity with RATE-XR can help researchers in the design and execution of studies within the guideline's scope. Given the challenge of reporting all required information into a single manuscript, authors may need to refer to other documents, such as study protocols, previous publications, and supplementary materials from digital repositories.

### Lessons Learned

The RATE-XR guideline represents the outcome of an extensive international consensus effort from a diverse and representative group of experts with a broad range of professional expertise and backgrounds. The high response rate and the remarkable level of engagement from stakeholders, along with the fact that 5 of the 7 most productive authors of XR research in psychology or medicine were willing to be included in the Steering Group, underscore the necessity for comprehensive reporting guidance in the early-phase clinical evaluation of XR applications [2]. This growing recognition highlights the increasing importance attributed to thorough clinical evaluation as a cornerstone for the effective implementation of XR technologies. The development of this reporting guideline was shaped by the Steering Group's belief that the use of XR-related health care applications will continue to expand, with an increasing requirement for high-quality, comprehensive, consistent, and generalizable reporting of early-phase evaluations.

The RATE-XR guideline is a pioneering endeavor, being the world's first reporting guideline specifically tailored to medical or psychology research involving XR-related applications. We focused on the early-phase clinical evaluation of XR-related applications, as existing guidelines insufficiently represented the essential reporting items for this type of research. Studies on late-phase evaluation often have the option to adhere to more general reporting guidelines, such as CONSORT for randomized controlled trials. However, we acknowledge that beyond this initial guideline, there is merit in further developing XR-specific extensions for existing guidelines. Thus, our efforts mark the crucial first step in harmonizing XR research in the health care sector. Beyond its primary aim, the RATE-XR guideline may also serve as a compass for authors, guiding them in study design, protocol development, and the registration of early-phase studies involving XR applications.

To attract experts with diverse backgrounds across the health care field, we published a call for contributions in the form of a correspondence paper. In this publication, we mainly focused on the terminology “virtual reality,” although we already had the more inclusive terminology “XR” in scope. During extensive deliberations during the Delphi rounds and after discussion within the Steering Group and Consensus Group meetings, we decided to use the more inclusive term XR instead of VR. This change allows the guideline to cover a broader range of applications, including augmented and MR, as well as any future immersive technologies. We concluded that XR terminology better represents all these applications, and all eventually will have to undertake equal and similar steps during early-stage evaluation and research. The decision to change the project's name from RATE-VR to RATE-XR reflects the guideline's adaptability and commitment to serving a diverse spectrum of applications beyond just focusing on VR alone.

The Delphi process, while invaluable for achieving consensus on guideline development, presented challenges such as maintaining a high follow-up rate among participants and reconciling diverse expert opinions. Our approach to addressing these challenges involved adaptive communication strategies and fostering an environment conducive to open dialogue, which were instrumental in enhancing participant engagement and consensus quality. These experiences provide key learnings that could inform similar guideline development efforts in emerging research fields.

Throughout the guideline's development, several topics generated more dynamic discussions than others, leading to a number of critical decisions. First, a discussion concerned the depth of information required about those administering XR applications. Some participants advocated for gathering baseline researcher or provider characteristics and offering detailed accounts of their training and qualifications. Given the novelty of the technology, the consensus was that mentioning that application providers had sufficient training was sufficient with no specific detailed requirement or baseline demographics unless deemed pivotal for study outcomes. In concordance, a recent review concluded that XR providers need training to improve adoption, which can be achieved using a variety of training programs, strategies, or educational resources, and that there is no minimum amount or golden standard of XR training [41].

Second, the deliberation on XR-specific outcomes, including factors such as acceptability, usability, user experiences, immersiveness, and cybersickness or other negative side-effects, sparked lengthy discussions among participants. While the importance of reporting cybersickness and safety-related aspects in early-phase evaluations was unanimously acknowledged for building trust within the research community, participants recognized the challenges of encompassing all XR-specific outcomes comprehensively in every article. Consequently, it was agreed that cybersickness and safety should be mandatory reporting items, while other XR-specific outcomes should be considered optional, acknowledging the difficulty in fully addressing all these outcomes in every manuscript. This approach allows researchers the flexibility to focus on the most relevant outcomes for their specific studies.

Third, intensive debates centered around the level of detail necessary when describing XR application hardware, software, and development processes in the RATE-XR guideline. While some argued for comprehensive and mandatory disclosure, others championed flexibility. Ultimately, agreement was reached to consolidate these aspects into a single section in the guideline, with the Explanation & Elaboration note providing guidance on what to include and how to effectively report them.

Fourth, the need for items on randomization in the current guideline was discussed. Participants felt that most early-phase evaluations are seldom randomized and acknowledged that if a study has a randomized design, adhering to established guidelines such as CONSORT would be more appropriate than duplicating information in the RATE-XR guideline. It was agreed that the guideline should not delve into specific items related to randomization, as it would be more beneficial for researchers to consult CONSORT and ensure consistency in reporting across various study designs. A similar consideration and strategy was recently adopted in the DECIDE-AI guideline to prevent a too exhaustive reporting checklist in the early stage of innovative technology development [13].

Fifth, the topic of blinding and the utilization of control groups triggered significant debate during the consensus process. Participants recognized the inherent challenges of blinding in XR studies due to the immersive nature of the technology. While some argued that specific items on blinding-related items should be included, others emphasized the challenges in doing so effectively. Furthermore, in alignment with the guideline's aim to avoid duplicating items covered by study design–specific guidelines, the decision was made to omit the item on blinding. Nevertheless, control groups were deemed valuable for comparison purposes, resulting in the inclusion of items that address the presence or absence of control groups and outline their characteristics as essential components within the guideline.

Sixth, discussions occurred regarding the necessity of justifying the sample sizes in the early-phase clinical assessment of XR applications. Participants held differing perspectives as to whether a formal sample size calculation should be mandated for all research types within the RATE-XR guideline. Ultimately, it was determined that while it is essential to provide some form of justification for the chosen sample size, not all research designs necessitate a formal sample size calculation. This decision recognizes the diversity of study designs and acknowledges that certain types of early-phase evaluations may have inherent limitations that preclude the use of traditional sample size calculations. Nevertheless, authors are strongly encouraged to provide a rationale and justification for their chosen sample size. This proactive step enhances transparency within the research process, enabling readers to assess the study's reliability of its findings.

Lastly, discussions concerning the inclusion of standardized statements such as data protection, ethics approval, and data and code availability were extensive. While their significance was acknowledged, the consensus was that most journals already require authors to address these elements in their manuscripts. Therefore, specific items related to these aspects were omitted from the RATE-XR guideline, as they are comprehensively covered by existing publication requirements and publication guidelines.

In conclusion, the RATE-XR guideline is a pioneering effort in facilitating comprehensive and standardized reporting in the early-phase clinical evaluation of XR applications. Its development involved extensive expert-informed debates and critical decisions that aim to ensure its purpose as a valuable resource for researchers while maintaining its adaptability to a dynamic and evolving field. We anticipate that this guideline will foster transparency, enhance the quality of reporting, and ultimately contribute to the responsible and effective integration of XR technologies in health care and related fields. Furthermore, we encourage the development of XR-specific extensions for existing guidelines to further advance the harmonization of XR research practices.

## Appendix

Multimedia Appendix 1 The RATE-XR (reporting for the early-phase clinical evaluation of applications using extended reality) qualitative study guideline.

## Abbreviations

AI: artificial intelligence
AR: augmented reality
CONSORT: Consolidated Standards of Reporting Trials
EQUATOR: Enhancing the Quality and Transparency of Health Research
MR: mixed reality
OSF: Open Science Framework
RATE-XR: reporting for the early-phase clinical evaluation of applications using extended reality
SPIRIT: Standard Protocol Items: Recommendations for Interventional Trials
STROBE: Strengthening the Reporting of Observational Studies in Epidemiology
VR: virtual reality
XR: extended reality

## Data Availability

The datasets and necessary documents generated and analyzed during this study are available in Multimedia Appendix 1 and the digital Open Science Framework RATE-XR platform [34]. Additionally, all study materials are available from the corresponding author upon reasonable request.
