# Supplementary material for: Reporting Guidelines for the Early-Phase Clinical Evaluation of Applications Using Extended Reality: RATE-XR Qualitative Study Guideline
Source: J Med Internet Res. 2024 Nov 29;26:e56790. doi: 10.2196/56790 (PMC11619188; doi:10.2196/56790)
Supplement: Multimedia Appendix 1 [file jmir_v26i1e56790_app1.docx]

**MULTIMEDIA APPENDIX 1**

Contents

[SUPPLEMENTARY FIGURES 5](#_Toc179447354)

[Suppl. Figure 1 - Item list(s) evolution (overview) 5](#_Toc179447355)

[Suppl. Figure 2 - Item list(s) evolution (per item) 6](#_Toc179447356)

[SUPPLEMENTARY TABLES 7](#_Toc179447357)

[Suppl. Table 1 - Composition of the Steering Group. 7](#_Toc179447358)

[Suppl. Table 2 - Demographics of Consensus group members. 8](#_Toc179447359)

[Suppl. Table 3 - Geographical distribution of Consensus Group members. 8](#_Toc179447360)

[Suppl. Table 4 - Stakeholder group affiliation of Consensus Group members. 8](#_Toc179447361)

[Suppl. Table 5 - Demographics of Delphi process participants. 8](#_Toc179447362)

[Suppl. Table 6 - Geographical distribution of Delphi process participants. 9](#_Toc179447363)

[Suppl. Table 7 - Stakeholder group affiliation of Delphi process participants. 9](#_Toc179447364)

[Suppl. Table 8 - Qualifications of Delphi process participants per stakeholder group. 9](#_Toc179447365)

[Suppl. Table 9 - Summary of the Consensus Meeting votes. 12](#_Toc179447366)

[SUPPLEMENTARY NOTES 14](#_Toc179447367)

[Suppl. Note 1 - Consensus process participants 14](#_Toc179447368)

[Suppl. Note 2 - Explanation & Elaboration of checklist items 19](#_Toc179447369)

[Title and Abstract - Item 1 (Title) 19](#_Toc179447370)

[Title and Abstract - Item I (Abstract) 19](#_Toc179447371)

[Introduction - Item 2 (Clinical Problem and Existing Evidence) 21](#_Toc179447372)

[Introduction - Item 3 (Introduction of the application) 21](#_Toc179447373)

[Introduction - Item II (Objectives) 22](#_Toc179447374)

[Methods and Analysis - Item III (Trial Design and Reporting) 23](#_Toc179447375)

[Methods and Analysis - Item IV (Trial Design and Reporting) 24](#_Toc179447376)

[Methods and analysis - Item 4 (Participants and Setting) 25](#_Toc179447377)

[Methods and Analysis - Item 5a (Participants and Setting) 25](#_Toc179447378)

[Methods and Analysis - Item 5b (Participants and Setting) 26](#_Toc179447379)

[Methods and Analysis - Item 6 (Intervention and Procedures) 27](#_Toc179447380)

[Methods and Analysis - Item 7 (Intervention and Procedures) 28](#_Toc179447381)

[Methods and Analysis - Item 8 (Intervention and Procedures) 29](#_Toc179447382)

[Methods and Analysis - Item V (Intervention and Procedures) 30](#_Toc179447383)

[Methods and Analysis - Item VI (Outcomes) 31](#_Toc179447384)

[Methods and Analysis - Item 9 (Outcomes) 32](#_Toc179447385)

[Methods and Analysis - Item VII (Sample Size) 33](#_Toc179447386)

[Methods and Analysis - Item VIII (Analysis) 34](#_Toc179447387)

[Methods and Analysis - Item IX (Protocol alterations) 35](#_Toc179447388)

[Results - Item X (Participants Flow and Recruitment) 35](#_Toc179447389)

[Results - Item XI (Baseline Data) 36](#_Toc179447390)

[Results - Item XII (Main Results) 37](#_Toc179447391)

[Results - Item 10 (Extended Reality and Human Factors) 38](#_Toc179447392)

[Results - Item 11 (Extended Reality and Human Factors) 39](#_Toc179447393)

[Results - Item 12 (Safety and Harms) 39](#_Toc179447394)

[Discussion and Conclusion - Item 13 (Generalizability and Impact) 40](#_Toc179447395)

[Discussion and Conclusion - Item 14 (Safety and Harms) 41](#_Toc179447396)

[Discussion and Conclusion - Item 15 (Ethics) 42](#_Toc179447397)

[Discussion and Conclusion - Item XIII (Strengths and Limitations) 42](#_Toc179447398)

[Discussion and Conclusion - Item 16 (Conclusion) 43](#_Toc179447399)

[Statements - Item XIV (Funding and Conflicts of Interest) 44](#_Toc179447400)

[Statements - Item 17 (Application) 45](#_Toc179447401)

# SUPPLEMENTARY FIGURES

## Suppl. Figure 1 - Item list(s) evolution (overview)

**
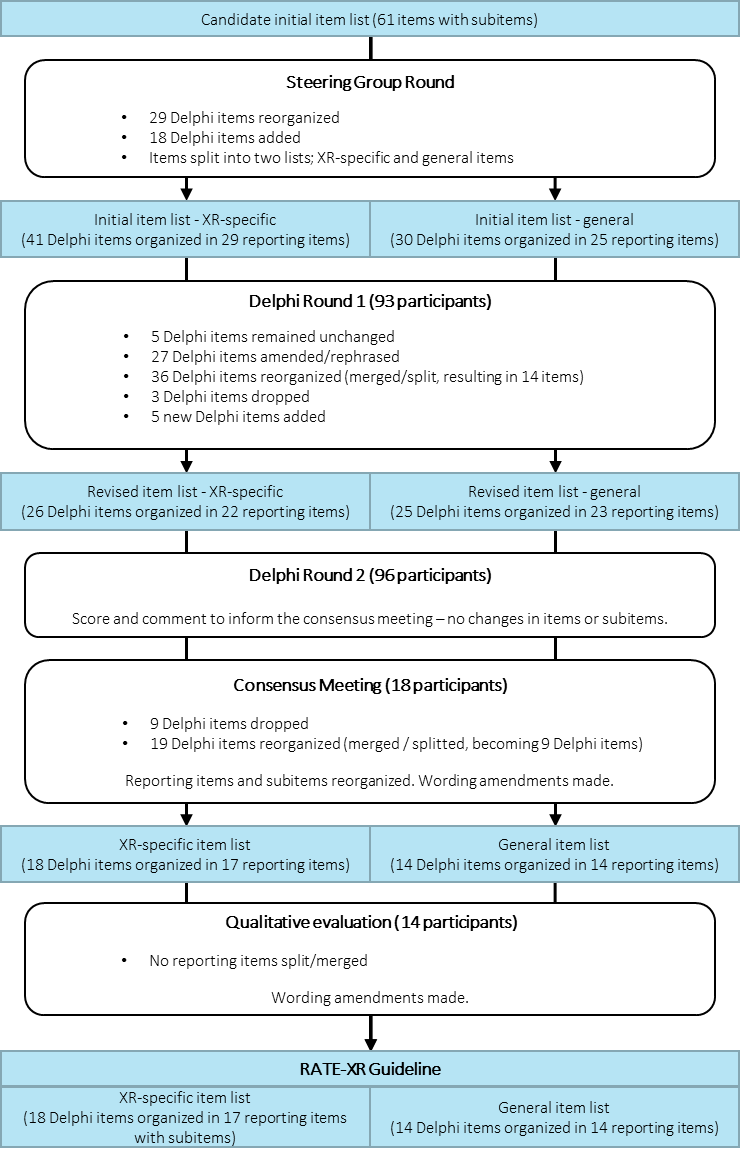
**

**Suppl. Figure 1:** Visual representation illustrating the item list(s) evolution during the guideline development process. Delphi items are the recommendations voted on during the Delphi rounds and discussed on during the consensus meeting; reporting items consist of either individual Delphi items or groupings of multiple ones, organized in a thematic manner.

## Suppl. Figure 2 - Item list(s) evolution (per item)
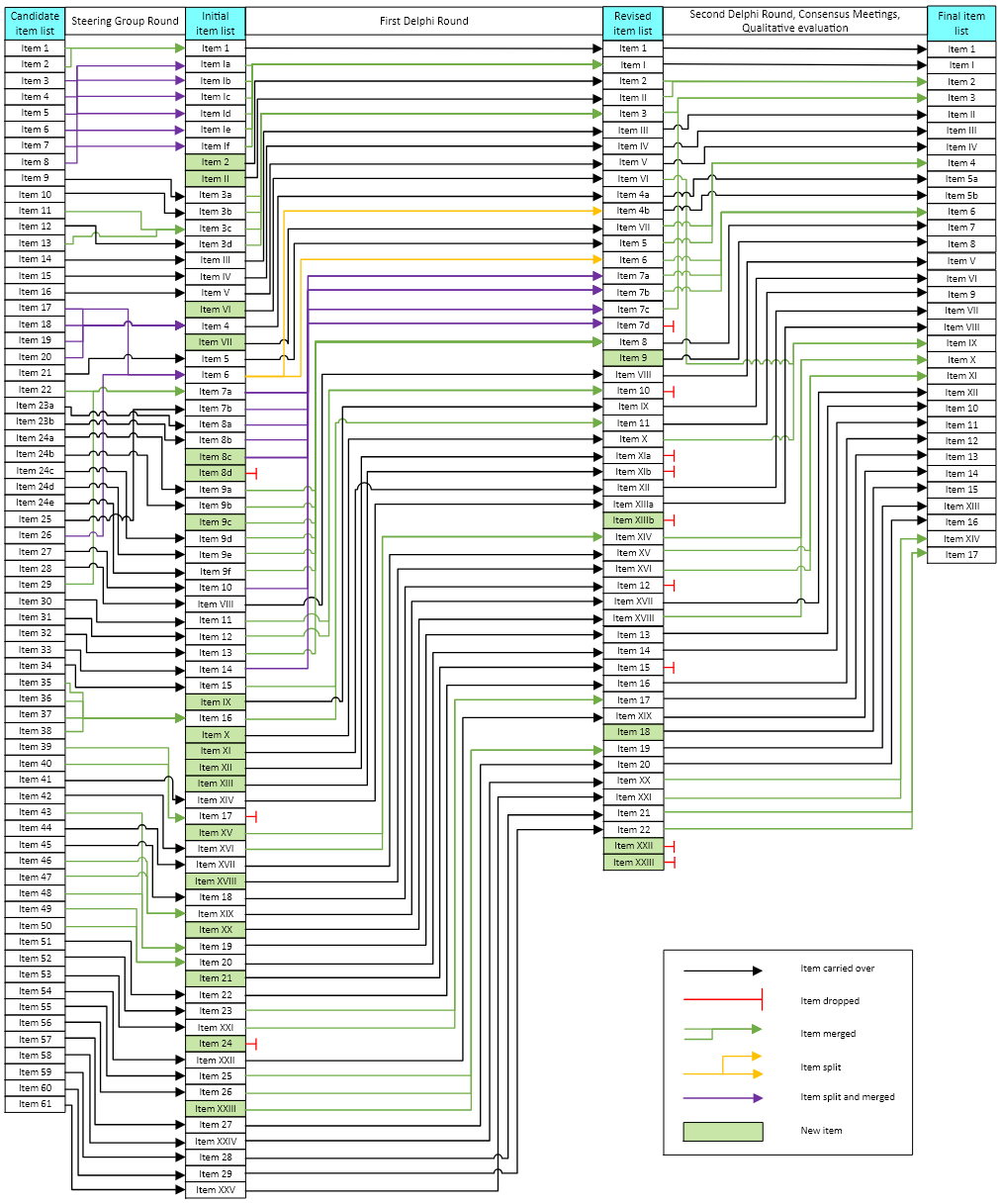


**Suppl. Figure 2:** Item list evolution per item. Items are listed in the same order as in the checklist, with XR-specific items being ordered by Arab numerals and generic items by Roman numerals. Black arrows are used for items that were carried over, the red line red denotes represent dropped items, green arrows are used for items that were merged, yellow arrows for items that were split during the revision of the item list, and green colored (boxes) items represent new items.

# SUPPLEMENTARY TABLES

| Suppl. Table 1 - Composition of the Steering Group. | | |
| --- | --- | --- |
| **Name** | **Affiliation** | **Stakeholder group** |
| Albert Skip Rizzo, PhD | Medical Virtual Reality Lab, University of Southern California Institute for Creative Technologies, Los Angeles, CA, USA | Researcher |
| Barbara O. Rothbaum, PhD | Emory University School of Medicine, Atlanta, GA, USA | Clinician  Researcher |
| Bart Geerts, MD, PhD, MSc, MBA | Healthplus.ai-R&D BV, Amsterdam, NL | Entrepreneur  Private sector representative |
| Brandon J. Birckhead, MD, MHDS | Department of Psychiatry and Behavioral Sciences, Johns Hopkins University School of Medicine, Baltimore, MD, USA | Clinician  Psychologist/Psychiatrist Researcher |
| Brenda K. Wiederhold, PhD, MBA, BCB, BCN | Virtual Reality Medical Center, San Diego, CA, USA | Clinical Health Psychologist Entrepreneur  Journal editor |
| Christian Jung, MD, PhD | Department of Cardiology, Pulmonology, and Vascular Medicine, Medical Faculty, University Hospital Düsseldorf, Heinrich-Heine-University Düsseldorf, Düsseldorf, DE  Cardiovascular Research Institute Düsseldorf (CARID), Medical Faculty and University Hospital of Düsseldorf, Heinrich-Heine-University Düsseldorf, Düsseldorf, DE | Clinician  Journal editor  Researcher |
| Cristina Botella, PhD | Department of Basic Psychology, Clinic, and Psychobiology of the University Jaume I, Castellón, ES  CIBER de Fisiopatología de la Obesidad y Nutrición (CIBEROBN), Instituto Salud Carlos III, Madrid, ES | Clinician  Psychologist/Psychiatrist  Researcher |
| Denzel L.Q. Drop, MD | Department of Intensive Care, Erasmus Medical Center, Rotterdam, NL | Clinician  Researcher |
| Diederik Gommers, MD, PhD | Department of Intensive Care, Erasmus Medical Center, Rotterdam, NL | Clinician  Hospital management  Researcher |
| Evert-Jan Wils, MD, PhD | Department of Intensive Care, Franciscus Gasthuis & Vlietland, Rotterdam, NL | Clinician  Researcher |
| Giuseppe Riva, PhD | Applied Technology for Neuro-Psychology Lab, IRCCS Istituto Auxologico Italiano, Milan, IT  Department of Psychology, Catholic University of the Sacred Heart, Milan, IT | Journal editor,  Psychologist/Psychiatrist Researcher |
| Jasper van Bommel, MD, PhD | Department of Intensive Care, Erasmus Medical Center, Rotterdam, NL | Clinician  Researcher |
| Johan H. Vlake, BSc | Department of Intensive Care, Erasmus Medical Center, Rotterdam, NL  Department of Intensive Care, Franciscus Gasthuis & Vlietland, Rotterdam, NL | Researcher |
| Lotty Hooft, PhD | Department of Epidemiology, Julius Center for Health Sciences and Primary Care, University Medical Center Utrecht, Utrecht, NL | Epidemiologist  Implementation specialist  Methodologist  Researcher |
| Michel E. van Genderen, MD, PhD | Department of Intensive Care, Erasmus Medical Center, Rotterdam, NL | Clinician  Researcher |
| O. Joseph Bienvenu, MD, PhD | Department of Psychiatry and Behavioral Sciences, Johns Hopkins University School of Medicine, Baltimore, MD, USA | Psychologist/Psychiatrist Researcher |
| Pietro Cipresso, PhD | Department of Psychology, University of Turin, Turin, IT  Applied Technology for Neuro-Psychology Lab, IRCCS Istituto Auxologico Italiano, Milan, IT | Computer scientists  Journal Editor  Methodologist  Researcher |

**Suppl. Table 1:** Composition of the Steering Group. Steering Group members are displayed alphabetically by their first names.

| Suppl. Table 2 - Demographics of Consensus group members. | | |
| --- | --- | --- |
| **Demographic** | | |
| Age, mean (SD) | | 45 (12) |
| Gender, female, n (%) | | 2 (11) |
| Published XR papers, n (%) | | 16 (89) |
|  | Number of publications on XR, median (IQR) | 10 (4 – 45) |
|  | Number of XR publications as first/second author, median (IQR) | 8 (3 – 23) |
| Involved in the development of XR applications, n (%) | | 15 (83) |
|  | Number of applications, median (IQR) | 2 (1 – 7) |
| **Suppl. Table 2:** Demographics of the Consensus Group members retrieved from the participation form. Total number of participants = 18. | | |

| Suppl. Table 3 - Geographical distribution of Consensus Group members. | | | |
| --- | --- | --- | --- |
| **Country** | **Number of participants (%)*** | **Country** | **Number of participants (%)*** |
| Netherlands | 4 (22) | Italy | 2 (11) |
| United States of America | 3 (17) | Canada | 1 (6) |
| Denmark | 2 (11) | Greece | 1 (6) |
| Spain | 2 (11) | United Kingdom | 1 (6) |
| Germany | 2 (11) |  |  |
| **Suppl. Table 3:** Geographical distribution of the Consensus Group members’ main working place. Total number of participants = 18. *Percentages are rounded, therefore the total sum is > 100%. | | | |

| Suppl. Table 4 - Stakeholder group affiliation of Consensus Group members. | | | |
| --- | --- | --- | --- |
| **Stakeholder group** | **Number of participants (%)** | **Stakeholder group** | **Number of participants (%)** |
| Clinicians | 12 (67) | Researchers | 16 (89) |
| Engineers/Computer scientists | 1 (6) | Ethicists | 1 (6) |
| Methodologists | 6 (33) | Patient representatives | 1 (6) |
| Statisticians | 2 (11) | Psychologists/psychiatrists | 4 (22) |
| Implementation experts | 1 (6) | Journal editors | 3 (17) |
| Entrepreneurs | 1 (6) | Allied health professionals | 2 (11) |
| Epidemiologists | 3 (17) |  |  |
| **Suppl. Table 4:** Self-reported stakeholder group affiliation(s) of the Consensus Group members. Total number of participants = 18. Each member could select multiple stakeholder group affiliations. | | | |

| Suppl. Table 5 - Demographics of Delphi process participants. | | |
| --- | --- | --- |
| **Demographic** | | |
| Age, mean (SD) | | 43 (12) |
| Gender, female, n (%) | | 48 (45) |
| Published XR papers, n (%) | | 79 (74) |
|  | Number of publications on XR, median (IQR) | 5 (2 – 19) |
|  | Number of XR publications as first/second author, median (IQR) | 4 (1 – 16) |
| Involved in the development of XR applications, n (%) | | 87 (81) |
|  | Number of applications, median (IQR) | 3 (2 – 4) |
| **Suppl. Table 5:** Demographics of the Delphi participants retrieved from the participation form. Total number of different participants over the two Delphi rounds = 107. | | |

| Suppl. Table 6 - Geographical distribution of Delphi process participants. | | | |
| --- | --- | --- | --- |
| **Country** | **Number of participants (%)*** | **Country** | **Number of participants (%)*** |
| Netherlands | 41 (38) | Canada | 4 (4) |
| USA | 21 (20) | Denmark | 4 (4) |
| Germany | 10 (9) | Belgium | 1 (1) |
| United Kingdom | 8 (7) | Greece | 1 (1) |
| Spain | 5 (5) | Hong Kong | 1 (1) |
| Italy | 5 (5) | Ireland | 1 (1) |
| Australia | 4 (4) | Turkey | 1 (1) |
| **Suppl. Table 6:** Geographical distribution of the Delphi participants’ main working place. Total number of different participants over the two Delphi rounds = 107. *Percentages are rounded, therefore the total sum is > 100%. | | | |
|  | | | |

| Suppl. Table 7 - Stakeholder group affiliation of Delphi process participants. | | | |
| --- | --- | --- | --- |
| **Stakeholder group** | **Number of participants (%)** | **Stakeholder group** | **Number of participants (%)** |
| Clinicians | 56 (53) | Policy makers/official institutional staff | 2 (2) |
| Engineers/Computer scientists | 7 (7) | Administrators/hospital management | 1 (1) |
| Methodologists | 14 (13) | Researchers | 88 (82) |
| Statisticians | 3 (3) | Ethicists | 2 (2) |
| Implementation specialists | 13 (12) | Private sector representatives | 5 (5) |
| Entrepreneurs | 12 (11) | Patient representatives | 1 (1) |
| Epidemiologists | 5 (5) | Funders | 1 (1) |
| Journal editors | 8 (8) | Psychologists/psychiatrists | 29 (27) |
| Allied health professionals | 9 (8) |  |  |
| **Suppl. Table 7:** Self-reported stakeholder group affiliation(s) of the Delphi participants. Total number of different participants over the two Delphi rounds = 107. Each participant could select multiple stakeholder group affiliations. | | | |

| Suppl. Table 8 - Qualifications of Delphi process participants per stakeholder group. | | | | |
| --- | --- | --- | --- | --- |
| **Clinicians (n = 56)** | | | |  |
|  | Years of experience, mean (SD) | | | 16 (10) |
|  | Setting, n (%) | | University hospital | 41 (73) |
|  |  | | Community hospital | 9 (16) |
|  |  | | Private clinic | 3 (5) |
| **Engineers/Computer scientists (n = 7)** | | | |  |
|  | Years of experience, mean (SD) | | | 17 (11) |
| **Methodologists (n = 14)** | | | |  |
|  | Years of experience, mean (SD) | | | 16 (9) |
|  | Involved in methodology of XR-related research, yes, n (%) | | | 11 (79) |
|  |  | Number of studies, median (IQR) | | 6 (3 – 18) |
|  | Involved in methodology of research on other innovative techniques, yes, n (%) | | | 12 (86) |
|  |  | Number of studies, median (IQR) | | 10 (5 – 34) |
|  | Involved in the development of other guidelines, yes, n (%) | | | 10 (71) |
|  |  | Number of guidelines, median (IQR) | | 2 (1 – 3) |
| **Statisticians (n = 3)** | | | |  |
|  | Years of experience, mean (SD) | | | 21 (9) |
|  | Involved in statistics of XR-related research, yes, n (%) | | | 2 (67) |
|  |  | Number of studies, median (IQR) | | 28 (17 – 39) |
|  | Involved in statistics of research on other innovative techniques, yes, n (%) | | | 3 (100) |
|  |  | Number of studies, median (IQR) | | 10 (8 – 45) |
| **Implementation Specialists (n = 13)** | | | |  |
|  | Years of experience, mean (SD) | | | 6 (5) |
|  | Involved in implementation of XR applications, yes, n (%) | | | 11 (85) |
|  |  | Number of applications, median (IQR) | | 3 (2 – 5) |
|  | Involved in implementation of applications using other innovative techniques, yes, n (%) | | | 5 (38) |
| **Entrepreneurs (n = 12)** | | | |  |
|  | Years of experience, mean (SD) | | | 8 (8) |
|  | Enterprise related to XR, yes, n (%) | | | 10 (83) |
|  | Enterprise related to other innovative techniques in healthcare, yes, n (%) | | | 8 (67) |
| **Epidemiologists (n = 5)** | | | |  |
|  | Years of experience, mean (SD) | | | 19 (12) |
|  | Involved in XR projects, yes, n (%) | | | 4 (100) |
|  |  | Number of projects, median (IQR) | | 5 (3 – 17) |
|  | Involved in development of other guidelines, yes, n (%) | | | 3 (75) |
|  |  | Number of guidelines, median (IQR) | | 2 (2 – 3) |
| **Journal Editors (n = 8)** | | | |  |
|  | Years of experience, mean (SD) | | | 9 (6) |
|  | Edited XR-related publications, yes, n (%) | | | 7 (88) |
|  |  | Number of publications, median (IQR) | | 50 (29 – 80) |
|  | Involved in development of other guidelines, yes, n (%) | | | 5 (63) |
|  |  | Number of guidelines, median (IQR) | | 2 (2 – 2) |
| **Allied Health Professionals (n = 9)** | | | |  |
|  | Years of experience, mean (SD) | | | 20 (13) |
|  | Involved in XR-related research, yes, n (%) | | | 7 (78) |
|  |  | Number of studies, median (IQR) | | 4 (2 – 6) |
| **Policy makers / official institutional staff (n = 2)** | | | |  |
|  | Years of experience, mean (SD) | | | 8 (4) |
|  | Involved in XR projects, yes, n (%) | | | 2 (100) |
|  |  | Number of projects, median (IQR) | | 6 (4 – 8) |
|  | Involved in the development of other guidelines, yes, n (%) | | | 2 (100) |
|  |  | Number of guidelines, median (IQR) | | 2 (1 – 2) |
| **Administrators/hospital management (n = 2)** | | | |  |
|  | Years of experience, mean | | | 5 |
| **Researchers (n = 88)** | | | |  |
|  | Years of experience, mean (SD) | | | 14 (10) |
|  | Involved in XR-related research, yes, n (%) | | | 81 (92) |
|  |  | Number of studies, median (IQR) | | 4 (2 – 10) |
|  | Involved in research on other innovative techniques, yes, n (%) | | | 65 (74) |
|  |  | Number of studies, median (IQR) | | 3 (2 – 10) |
| **Ethicists (n = 2)** | | | |  |
|  | Years of experience, mean (SD) | | | 11 (6) |
|  | Involved in XR projects, yes, n (%) | | | 2 (100) |
|  |  | Number of projects, median (IQR) | | 3 (2 – 3) |
| **Private Sector representatives (n = 5)** | | | |  |
|  | Years of experience, mean (SD) | | | 12 (10) |
|  | Involved in XR projects, yes, n (%) | | | 5 (100) |
|  |  | Number of projects, median (IQR) | | 10 (5 – 50) |
| **Patient Representatives (n = 1)** | | | |  |
|  | Experience with XR applications, yes, n (%) | | | 1 (100) |
| **Funders (n = 1)** | | | |  |
|  | Years of experience | | | 28 |
|  | Number of funded XR projects | | | 30 |
|  | Number of funded projects of other innovative techniques | | | 40 |
| **Psychologists / Psychiatrists (n = 29)** | | | |  |
|  | Years of experience, mean (SD) | | | 17 (10) |
|  | Uses XR applications in common practice, yes, n (%) | | | 15 (52) |
|  | Involved in XR-related research, yes, n (%) | | | 25 (86) |
|  |  | Number of studies | | 6 (2 – 12) |
| **Suppl. Table 8:** Self-reported qualifications of Delphi process participants per stakeholder group. Each stakeholder group were shown questions specific to their expertise. Total number of different participants over the two Delphi rounds = 107. | | | | |

| Suppl. Table 9 - Summary of the Consensus Meeting votes. | | | | | | | | |
| --- | --- | --- | --- | --- | --- | --- | --- | --- |
|  | **Delphi** | **Consensus meeting** | | | | | | |
|  | % of participants | Votes | | | % of votes^c^ | | Results | Arguments/Comments of the Consensus Group |
|  | Include^a^ | Include | Exclude | Blank^b^ | Include | Exclude |  |  |
| **Item 1** | 82 | 18 | 0 | 0 | 100 | 0 | Include | Emphasizing that the study is an initial clinical assessment of an XR application is essential, while the remaining details are discretionary. |
| **Item I** | 91 | 14 | 3 | 1 | 82 | 18 | Include | Should be restructured and be made more concise. Structured should be between parenthesis as not all journal allow structured abstracts. Not all items need to be mandatory. |
| **Item 2** | 90 | 18 | 0 | 0 | 100 | 0 | Include | Merge with item II. |
| **Item II** | 75 | 16 | 2 | 0 | 89 | 11 | Include | ‘Gold Standard’ should be avoided, replace with ‘most frequently used’ or ‘evidence-based’. Merge with item 2. |
| **Item 3** | 87 | 18 | 0 | 0 | 100 | 0 | Include | Prioritize hypotheses on potential effects and past application research; other details can be omitted. Merge with item 7c. |
| **Item III** | 99 | 18 | 0 | 0 | 100 | 0 | Include |  |
| **Item IV** | 86 | 17 | 0 | 1 | 100 | 0 | Include | Ethical approval is mandatory, protocol and study registration can be optional due to potential non-English protocols. |
| **Item V** | 90 | 18 | 0 | 0 | 100 | 0 | Include |  |
| **Item VI** | 76 | 18 | 0 | 0 | 100 | 0 | Include | Merge with item X. |
| **Item 4a** | 94 | 18 | 0 | 0 | 100 | 0 | Include |  |
| **Item 4b** | 67 | 16 | 1 | 1 | 94 | 6 | Include | Prioritize whether application users were trained, not the number and duration of training sessions. |
| **Item VII** | 72 | 16 | 1 | 1 | 94 | 6 | Include | Merge with item 5. |
| **Item 5** | 66 | 17 | 1 | 0 | 94 | 6 | Include | Merge with item VII. |
| **Item 6** | 79 | 17 | 1 | 0 | 94 | 6 | Include | Merge with item 7a & b and unify content, hardware and application setup details. |
| **Item 7a** | 88 | 16 | 1 | 1 | 94 | 6 | Include | Merge with 6 and 7b. |
| **Item 7b** | 90 | 16 | 1 | 1 | 94 | 6 | Include | Merge with 6 and 7a. |
| **Item 7c** | 77 | 15 | 2 | 1 | 88 | 12 | Include | Merge with item 3. |
| **Item 7d** | 88 | 12 | 6 | 0 | 67 | 33 | Exclude | Integrate this information into the preceding items; it should not be a standalone entry. |
| **Item 8** | 65 | 12 | 3 | 3 | 80 | 20 | Include |  |
| **Item 9** | 80 | 18 | 0 | 0 | 100 | 0 | Include |  |
| **Item VIII** | 93 | 17 | 1 | 0 | 94 | 6 | Include | If no control is utilized, provide a clear explanation for its absence. |
| **Item 10** | 72 | 12 | 5 | 1 | 71 | 29 | Exclude | Beyond the scope of the current guideline, too general. |
| **Item IX** | 93 | 18 | 0 | 0 | 100 | 0 | Include |  |
| **Item 11** | 87 | 17 | 1 | 0 | 94 | 6 | Include |  |
| **Item X** | 81 | 15 | 2 | 1 | 88 | 12 | Include | Merge with item VI. |
| **Item XIa** | 78 | 14 | 4 | 0 | 78 | 22 | Exclude | For randomized controlled trials, authors must follow the CONSORT guidelines. |
| **Item XIb** | 86 | 13 | 4 | 1 | 76 | 24 | Exclude | For randomized controlled trials, authors must follow the CONSORT guidelines. |
| **Item XII** | 79 | 16 | 1 | 1 | 94 | 6 | Include |  |
| **Item XIIIa** | 93 | 17 | 0 | 1 | 100 | 0 | Include |  |
| **Item XIIIb** | 73 | 13 | 4 | 1 | 76 | 24 | Exclude | Dropped. Covered in items VI and X. |
| **Item XIV** | 84 | 18 | 0 | 0 | 100 | 0 | Include | Merge with item XVIII. |
| **Item XV** | 88 | 18 | 0 | 0 | 100 | 0 | Include | Merge with item XVI. |
| **Item XVI** | 78 | 13 | 2 | 3 | 87 | 13 | Include | Merge with item XV. |
| **Item 12** | 60 | 9 | 8 | 1 | 53 | 47 | Exclude | If the methods mention training for application users, additional details about their characteristics are unnecessary. |
| **Item XVII** | 94 | 18 | 0 | 0 | 100 | 0 | Include |  |
| **Item XVIII** | 95 | 17 | 1 | 0 | 94 | 6 | Include | Merge with item XIV. |
| **Item 13** | 87 | 17 | 0 | 1 | 100 | 0 | Include |  |
| **Item 14** | 91 | 15 | 2 | 1 | 88 | 12 | Include |  |
| **Item 15** | 66 | 11 | 5 | 2 | 69 | 31 | Exclude | Include them only if they are primary or secondary outcomes; a distinct item is unnecessary. |
| **Item 16** | 91 | 16 | 2 | 0 | 89 | 11 | Include |  |
| **Item 17** | 86 | 17 | 0 | 1 | 100 | 0 | Include |  |
| **Item XIX** | 87 | 18 | 0 | 0 | 100 | 0 | Include |  |
| **Item 18** | 73 | 13 | 3 | 2 | 81 | 19 | Include | Also current considerations, include risks and benefits. |
| **Item 19** | 93 | 17 | 1 | 0 | 94 | 6 | Include | Address strengths. Barriers for the use/implementation of the application are not study limitations and should be covered in item 17. |
| **Item 20** | 96 | 14 | 3 | 1 | 82 | 18 | Include | Safety and harms should not be named specifically. |
| **Item XX** | 86 | 17 | 1 | 0 | 94 | 6 | Include | Any funding source should be stated. Merge with item XXI. |
| **Item XXI** | 94 | 15 | 3 | 0 | 83 | 17 | Include | Merge with item XX. |
| **Item 21** | 87 | 16 | 2 | 0 | 89 | 11 | Include | Merge with item 22. |
| **Item 22** | 74 | 14 | 2 | 2 | 88 | 12 | Include | Merge with item 21. |
| **Item XXII** | 93 | 8 | 10 | 0 | 44 | 56 | Exclude | Integrate this information into the methods items; it should not be a standalone entry. |
| **Item XXIII** | 73 | 13 | 5 | 0 | 72 | 18 | Exclude | Is usually required by the journal, redundant to be part of the RATE-XR recommendations. |
| **Suppl. Table 9:** Summary of the Consensus Meetings votes and results. ^a^ A mean score ≥ 70% was defined as a recommendation to include. ^b^ Including abstentions. ^C^ Only considering non-blank votes. | | | | | | | | |

# SUPPLEMENTARY NOTES

## Suppl. Note 1 - Consensus process participants

RATE-XR Steering Group (n=17)

Albert Skip Rizzo (Medical Virtual Reality Lab, University of Southern California Institute for Creative Technologies, Los Angeles, CA, USA); Barbara O. Rothbaum (Emory University School of Medicine, Atlanta, GA, USA); Bart Geerts (Healthplus.ai-R&D BV, Amsterdam, NL); Brandon J. Birckhead (Department of Psychiatry and Behavioral Sciences, Johns Hopkins University School of Medicine, Baltimore, MD, USA); Brenda K. Wiederhold (Virtual Reality Medical Centers, San Diego, CA, USA); Christian Jung (Department of Cardiology, Pulmonology, and Vascular Medicine, Medical Faculty, University Hospital Düsseldorf, Heinrich-Heine-University Düsseldorf, Düsseldorf, DE, and Cardiovascular Research Institute Düsseldorf (CARID), Medical Faculty and University Hospital of Düsseldorf, Heinrich-Heine-University Düsseldorf, Düsseldorf, DE); Cristina Botella (Department of Basic Psychology, Clinic, and Psychobiology of the University Jaume I, Castellón, and CIBER de Fisiopatología de la Obesidad y Nutrición (CIBEROBN), Instituto Salud Carlos III, Madrid, ES); Denzel L. Q. Drop (Department of Intensive Care, Erasmus Medical Center, Rotterdam, NL); Diederik Gommers (Department of Intensive Care, Erasmus Medical Center, Rotterdam, NL); Evert-Jan Wils (Department of Intensive Care, Franciscus Gasthuis & Vlietland, Rotterdam, NL); Giuseppe Riva (Applied Technology for Neuro-Psychology Lab, IRCCS Istituto Auxologico Italiano, Milan, IT, and Department of Psychology, Catholic University of the Sacred Heart, Milan, IT); Jasper van Bommel (Department of Intensive Care, Erasmus Medical Center, Rotterdam, NL); Johan H. Vlake (Department of Intensive Care, Erasmus Medical Center, Rotterdam, NL, and Department of Intensive Care, Franciscus Gasthuis & Vlietland, Rotterdam, NL); Lotty Hooft (Department of Epidemiology, Julius Center for Health Sciences and Primary Care, University Medical Center Utrecht, Utrecht, NL); Michel E. van Genderen (Department of Intensive Care, Erasmus Medical Center, Rotterdam, NL); O. Joseph Bienvenu (Department of Psychiatry and Behavioral Sciences, Johns Hopkins University School of Medicine, Baltimore, MD, USA); Pietro Cipresso (Department of Psychology, University of Turin, Turin, IT, and Applied Technology for Neuro-Psychology Lab, IRCCS Istituto Auxologico Italiano, Milan, IT).

Delphi participants of both Delphi rounds (n=107)

Aisling Flynn (School of Nursing and Midwifery, University of Galway, Galway, IRE); Albert Skip Rizzo (Medical Virtual Reality Lab, University of Southern California Institute for Creative Technologies, Los Angeles, CA, USA); Alice Chirico (Catholic University of the Sacred Heart of Milan); Amir H. Sadeghi (Department of Cardiothoracic Surgery, Erasmus Medical Center, Rotterdam, NL); Andrea Gaggioli (Research Center in Communication Psychology (PSICOM), Catholic University of the Sacred Heart, Milan, IT, and IRCCS Istituto Auxologico Italiano, Milan, IT); Andrea S. Won (Department of Communication, Cornell University, Ithaca, NY, USA); Annelotte P. van Haaps (Department of Reproductive Medicine, Amsterdam University Medical Centers, location VUmc, Amsterdam, NL, and Amsterdam Reproduction and Development Research Institute, Amsterdam, NL); Azucena Garcia-Palacios (Department of Basic Psychology, Clinical Psychology and Psychobiology, Universitat Jaume I, Castellón, ES); Barbara O. Rothbaum (Emory University School of Medicine, Atlanta, GA, USA); Bart Geerts (Healthplus.ai-R&D BV, Amsterdam, NL); Beate Dejaco (Research Group Musculoskeletal Rehabilitation, HAN University of Applied Sciences, Nijmegen, NL, and Department of Surgery, Radboud University Medical Center, Nijmegen, NL); Bram Dierckx (Department of child and adolescent psychiatry/psychology, Erasmus Medical Center, Rotterdam, NL); Brandon J. Birckhead (Department of Psychiatry and Behavioral Sciences, Johns Hopkins University School of Medicine, Baltimore, MD, USA); Brenda K. Wiederhold ( Virtual Reality Medical Center, San Diego, CA, USA); Carsten Finke (Department of Neurology, Charité - Universitätsmedizin Berlin, Berlin, DE); Catheleine van Driel (Department of Psychiatry, University Medical Center Groningen, Groningen, NL); Chris N. W. Geraets (Department of Psychiatry, University Medical Center Groningen, Groningen, NL); Christian Jung ( Department of Cardiology, Pulmonology, and Vascular Medicine, Medical Faculty, University Hospital Düsseldorf, Heinrich-Heine-University Düsseldorf, Düsseldorf, DE, and Cardiovascular Research Institute Düsseldorf (CARID), Medical Faculty and University Hospital of Düsseldorf, Heinrich-Heine-University Düsseldorf, Düsseldorf, DE); Christopher Eccleston (The University of Bath, Bath, UK); Christopher R. Madan (School of Psychology, University of Nottingham, Nottingham, UK); Clarine van Oel (Faculty of Architecture and the Built Environment, Delft University of Technology, Delft, NL); Constantinos Panayi; Cristina Botella (Department of Basic Psychology, Clinic, and Psychobiology of the University Jaume I, Castellón, and CIBER de Fisiopatología de la Obesidad y Nutrición (CIBEROBN), Instituto Salud Carlos III, Madrid, ES); Darcy Ummels (Department of Rehabilitation Medicine, Maastricht University, Maastricht, NL); Dave Thomas (Office of Research on Women’s Health (ORWH), Office of the Director, National Institutes of Health, Democracy II, Bethesda, MD, USA); Debra L. Safer (Department of Psychiatry and Behavioral Scienes, Stanford University School of Medicine, Stanford, USA); Elsbeth Zandee (Intensive Outpatient Treatment Unit, GGZ Delfland, Delft, NL); Emil R. Høeg (Department of Architecture, Design and Media Technology, The Technical Faculty of IT and Design, Copenhagen SV, DK); Evert-Jan Wils (Department of Intensive Care, Franciscus Gasthuis & Vlietland, Rotterdam, NL); Floris van der Breggen (SyncVR Medical, Rotterdam, NL); Geert-Jan van Geffen (Department of Anesthesiology, Pain and Palliative Care, Radboud University Medical Center, Nijmegen, NL); Gido A. Hakvoort (Research group IT Innovations in Healthcare, Windesheim University of Applied Sciences, Zwolle, NL); Giulia Corno (Loricorps research unit, Centre de recherche de l'Institut Universitaire en santé Mentale de Montréal, Montreal, CA); Giuseppe Riva ( Applied Technology for Neuro-Psychology Lab, IRCCS Istituto Auxologico Italiano, Milan, IT, and Department of Psychology, Catholic University of the Sacred Heart, Milan, IT); Hadi Hosseini (Stanford University, Stanford, California, USA); Hafize Demirci (Department of Surgery, Amsterdam University Medical Centers, location University of Amsterdam, Amsterdam, NL); Hanne Konradsen (Herlev and Gentofte University Hospital, University of Copenhagen, Copenhagen, DK); Henry Xiang (Department of Pediatrics, The Ohio State University College of Medicine, Columbus, OH, USA); Jan D. Rölfing (Department of Orthopaedics, Aarhus University Hospital, Aarhus, DK, and Corporate HR, MidtSim, Aarhus, DK); Jan Gödeke (Pediatric Surgery, LMU Medical Center, LMU Munich, Munich, DE); Jasper van Bommel (Department of Intensive Care, Erasmus Medical Center, Rotterdam, NL); Jennifer N. Stinson (Research Institute, The Hospital for Sick Children, Toronto, CA); Jeroen Legerstee (Department of child and adolescent psychiatry/psychology, Erasmus Medical Center, Rotterdam, NL); Johan H. Vlake (Department of Intensive Care, Erasmus Medical Center, Rotterdam, NL, and Department of Intensive Care, Franciscus Gasthuis & Vlietland, Rotterdam, NL); Joost Huiskens (Account Executive Healthcare Microsoft, NL); Jordan Tsigarides (Norwich Medical School, University of East Anglia, Norwich, UK); Jose F. Costa (Innovation and projects, Badalona Serveis Assistencials, Badalona, ES); Juana M. Bretón-López (Department of Basic and Clinical Psychology and Psychobiology, Area of Personality, Psychological Assessment and Treatments, Universitat Jaume I, Castellón, ES); Karamveer Narang (Virtual Reality in Medicine and Surgery, vrims.net, UK); Karin Valkenet (Department of Physiotherapy Sciences, University Medical Center Utrecht, Utrecht, NL); Kim D. Bullock (Department of Psychiatry & Behavioral Sciences, Stanford University School of Medicine, Stanford, CA, USA); Kimmy Rosielle (Department of Obstretrics and Gynaecology, Amsterdam University Medical Center, VUmc, Amsterdam, NL); Krista Hoek (Department of Anesthesiology and Intensive Care, Leiden University Medical Center, Leiden, NL); Lars Riedemann (Heidelberg University Hospital, Heidelberg, DE); Line K. Pedersen (Department of Orthopaedic Surgery, Aarhus University Hospital, Aarhus, DK); Loes Bulle-Smid (Research group IT innovations in Healthcare, Windesheim University of applied sciences, Zwolle, NL); Lonneke M. Staals (Department of Anesthesiology, Erasmus Medical Center and Sophia Children's Hospital, Rotterdam, NL); Lotty Hooft (Department of Epidemiology, Julius Center for Health Sciences and Primary Care, University Medical Center Utrecht, Utrecht, NL); Kemal Kuscu (Department of Psychiatry and KARMA-Lab, Koc University, Istanbul, TR); Mareine G. T. Koornneef (GGZ Rivierduinen, NL); Margaux Sageot (Department of Psychology, University Psychiatric Center, Kortenberg, BE); Margot D. Paul (Department of Psychiatry and Behavioral Sciences, Stanford University School of Medicine, Stanford, USA); Maria Bajwa (Adjunct Faculty, Health Professions Education, MGH Institution of Health Professions, Boston, USA); Mariju F. Baluyot (Department of Emergency Medicine, Division of Pediatrics, Division of Simulation, Indiana University, Indianapolis, USA); Marlies P. Schijven (Department of Surgery, Amsterdam Gastroenterology and Metabolism, Amsterdam Public Health, Digital Health, Amsterdam University Medical Centers, University of Amsterdam, Amsterdam, NL); Martine J. van Bennekom (GGZ Delfland, Delft, NL); Melissa L. Morris (Dr. Pallavi Patel College of Healthcare Sciences, Nova Southeastern University, Ft. Lauderdale, FL, USA); Merel A. Oskam (VUmc School of Medical Sciences, Amsterdam, NL); Merlijn Smits (Department of Industrial design, Saxion University of Applied Sciences, Enschede, NL)); Michael Gaebler (Department of Neurology, Max Planck Institute for Human Cognitive and Brain Sciences, Leipzig, DE); Michel E. van Genderen (Department of Intensive Care, Erasmus Medical Center, Rotterdam, NL); Mienke Rijsdijk (Department of Anesthesiology, University Medical Center Utrecht, Utrecht, NL); Njin-Zu Chen (DECB Cognitive Engineering, Philips Research, Eindhoven, NL); Omar Aly (Department of Colorectal Surgery, Southampton General Hospital, Southampton, UK); O. Joseph Bienvenu (Department of Psychiatry and Behavioral Sciences, Johns Hopkins University School of Medicine, Baltimore, MD, USA); Panagiotis Kourtesis (Department of Psychology, Marican College of Greece, Athens, GR, and Department of Psychology, National and Kapodistrian University of Athens, Athens, GR, and Department of Psychology, University of Edinburgh, Edinburgh, UK); Philipp Kellmeyer (Department of Neurosurgery, University of Freiburg - Medical Center, Freiburg im Breisgau, DE); Pietro Cipresso (Department of Psychology, University of Turin, Turin, IT, and Applied Technology for Neuro-Psychology Lab, IRCCS Istituto Auxologico Italiano, Milan, IT); Les Posen (Independent Practitioner); Rachel Reeves (Northern Health & Social Care Trust, IE); Rami A. Ahmed (Department of Simulation, Indiana University School of Medicine, Indianapolis, USA); Raphael R. Bruno (Department of Cardiology, Pulmonology, and Vascular Medicine, Medical Faculty, University Hospital Düsseldorf, Heinrich-Heine-University Düsseldorf, Düsseldorf, DE); Rob J. E. M. Smeets (Department of Rehabilitation Medicine, Care and Public Health Research Institute (CAPHRI), Faculty of Health, Medicine & Life Sciences, Maastricht University, Maastricht, NL, and CIR Clinics in Revalidatie, Eindhoven, NL); Robbert Brouwer (SyncVR Medical, Rotterdam, NL); Robert J. Fine (International Virtual Reality and Healthcare Association (IVRHA), Washington, DC, USA); Robert M. Lundin (Institute for Mental and Physical Health and Clinical Translation (IMPACT), Deakin University, Geelong, AUS); Roderick F. van Beek (Department of Implementation, SyncVR Medical, Rotterdam, NL); Rosa M. Baños (Department of Personality, Assessment, and Psychological Treatments, Polibieinestar Institute, University of Valencia, Valencia, ES); Roselinde M. C. A. Pot-Kolder (Orygen National Centre of Excellence in Youth Mental Health, The University of Melbourne, Melbourne, AU); Sarah E. MacPherson (Department of Psychology, University of Edinburgh, Edinburgh, UK); Silvia Serino (Department of Psychology, Università of Milano-Bicocca, Milan, IT); Sophia Rekers (Department of Neurology, Charité-Universitätsmedizin Berlin, Berlin, DE, and Corporate Member of Freie Universität Berlin, Berlin, DE, and Humboldt-Universität zu Berlin, Berlin, DE); Srinivasan S. Pillay (Chief Medical Officer, Reulay, New York, USA); Stephan Krohn (Department of Neurology, Charité-Universitätsmedizin Berlin, Berlin, DE, and Berlin School of Mind and Brain, Humboldt-Universität zu Berlin, DE); Stéphane Bouchard (Department of Psychoeducation and Psychology, University of Québec in Outaouais, Gatineau, CA); Sulayman el Mathari (Department of Cardiothoracic Surgery, Amsterdam University Medical Center, Amsterdam, NL); Susan Persky (Social and Behavioral Research Branch, National Human Genome Research Institute, Bethesda, US); Syl Slatman (Musculoskeletal Rehabilitation Research Group, School for Allied Health, HAN University of Applied Sciences, Nijmegen, NL, and Biomedical Signals and Systems Group, Faculty of Electrical Engineering, Mathematics and Computer Science, University of Twente, Enschede, NL); Synthia Guimond (Department of Psychoeducation and Psychology, University of Québec in Outaouais, Gatineau, CA, and Department of Psychiatry, The Royal’s of Mental Health Research, University of Ottawa, Ottawa, CA); Thomas J. Caruso (Department of Anesthesiology, Perioperative, and Pain Medicine, Stanford University School of Medicine, USA); Thomas Wolbers (German Center for Neurodegenerative Diseases, Göttingen, DE); Tjitske D. Groenveld (Department of Surgery, Radboud University Medical Center, Nijmegen, NL); Tobias Loetscher (Department of Cognitive Ageing and Impairment Neurosciences, University of South Australia, Adelaide, AU); Tonnie Staring (Psychosis Department, Psychologist Netherlands Mental Health Institute, Utrecht, NL); Vishnunarayan G. Prabhu (Industrial and Systems Engineering, University of North Carolina at Charlotte, Charlotte, NC, USA); Wim Veling (Department of Psychiatry, University Medical Center Groningen, University of Groningen, Groningen, NL); Winnie W. S. Mak (Department of Psychology, The Chinese University of Hong Kong, Shatin, HK, CN).

Consensus meeting members (n= 18)

*With voting rights:* Brandon J. Birckhead (**June 15^th^**) (Department of Psychiatry and Behavioral Sciences, Johns Hopkins University School of Medicine, Baltimore, MD, USA); Christian Jung (**June 12^th^**) (Department of Cardiology, Pulmonology, and Vascular Medicine, Medical Faculty, University Hospital Düsseldorf, Heinrich-Heine-University Düsseldorf, Düsseldorf, DE, and Cardiovascular Research Institute Düsseldorf (CARID), Medical Faculty and University Hospital of Düsseldorf, Heinrich-Heine-University Düsseldorf, Düsseldorf, DE); Cristina Botella (**June 15^th^**) (Department of Basic Psychology, Clinic, and Psychobiology of the University Jaume I, Castellón, and CIBER de Fisiopatología de la Obesidad y Nutrición (CIBEROBN), Instituto Salud Carlos III, Madrid, ES); Denzel L. Q. Drop (**June 12^th^**) (Department of Intensive Care, Erasmus Medical Center, Rotterdam, NL); Emil Rosenlund Høeg (**June 15^th^**) (The Faculty of IT and Design, Aalborg University Copenhagen, Aalborg, DK); Evert-Jan Wils (**June 12^th^**) (Department of Intensive Care, Franciscus Gasthuis & Vlietland, Rotterdam, NL): Giuseppe Riva (**June 12^th^**) (Applied Technology for Neuro-Psychology Lab, IRCCS Istituto Auxologico Italiano, Milan, IT, and Department of Psychology, Catholic University of the Sacred Heart, Milan, IT); Henry Xiang (**June 15^th^**) (Department of Pediatrics, The Ohio State University College of Medicine, Columbus, OH, USA); Johan H. Vlake (**June 12^th^**) (Department of Intensive Care, Erasmus Medical Center, Rotterdam, NL and Department of Intensive Care, Franciscus Gasthuis & Vlietland, Rotterdam, NL); Jose F. Costa (**June 13^th^**) (Innovation and projects, Badalona Serveis Assistencials, Badalona, ES); Line Keldgaard (**June 13^th^**) (Aarhus University Hospital, Aarhus, DK); Michel E. van Genderen (**June 15^th^**) (Department of Intensive Care, Erasmus Medical Center, Rotterdam, NL); O. Joseph Bienvenu (**June 15^th^**) (Department of Psychiatry and Behavioral Sciences, Johns Hopkins University School of Medicine, Baltimore, MD, USA); Panagiotis Kourtesis (**June 15^th^**) (Department of Psychology, Marican College of Greece, Athens, GR, and Department of Psychology, National and Kapodistrian University of Athens, Athens, GR, and Department of Psychology, University of Edinburgh, Edinburgh, UK); Constantinos Panayi (**June 12^th^**); Phillip Kellmeyer (**Jun 13^th^**) (Department of Neurosurgery, University of Freiburg - Medical Center, Freiburg im Breisgau, DE); Pietro Cipresso (**June 15^th^**) (Department of Psychology, University of Turin, Turin, IT, and Applied Technology for Neuro-Psychology Lab, IRCCS Istituto Auxologico Italiano, Milan, IT); Stéphane Bouchard (**June 13^th^**) (Department of Psychoeducation and Psychology, University of Québec in Outaouais, Gatineau, CA).

*Chair:* Michel E. van Genderen (Department of Intensive Care, Erasmus Medical Center, Rotterdam, NL; **June 12^th^**); Johan H. Vlake (Department of Intensive Care, Erasmus Medical Center, Rotterdam, NL, and Department of Intensive Care, Franciscus Gasthuis & Vlietland, Rotterdam, NL; **June 13^th^, June 15^th^**).

*Observers:* Johan H. Vlake (Department of Intensive Care, Erasmus Medical Center, Rotterdam, NL, and Department of Intensive Care, Franciscus Gasthuis & Vlietland, Rotterdam, NL; **June 12th**); Denzel L. Q. Drop (Department of Intensive Care, Erasmus Medical Center, Rotterdam, NL; **June 13^th^, June 15^th^**).

Qualitative evaluation experts (n= 14 )

Debra L. Safer (Department of Psychiatry and Behavioral Sciences, Stanford University School of Medicine, Stanford, USA); Felix J. Hüttner (Department of General and Visceral surgery, Ulm University Hospital, Ulm, DE); Hanne Konradsen (Herlev and Gentofte University Hospital, University of Copenhagen, Copenhagen, DK); Ivan Phelan (Sheffield Hallam University, Sheffield, UK); Jeremy Bailenson (Stanford University, Stanford, California, USA); Jiabin Shen (University of Massachusetts Lowell, Lowell, USA); Maria Matsangidou (CYENS Centre of Excellence, Nicosia, Cyprus); Marie-Madlen Jeitziner (Department of Intensive Care Medicine, University Hospital Bern, Inselspital, University of Bern, Bern, CH); Matthew Browning (Clemson University, Clemson, USA); Pablo Campo-Prieto (University of Vigo and HealthyFit Research Group, Vigo, ES); Raphael Bruno (Department of Cardiology, Pulmonology, and Vascular Medicine, Medical Faculty, University Hospital Düsseldorf, Heinrich-Heine-University Düsseldorf, Düsseldorf, DE); Thomas Sauter (University of Bern, Bern, CH); Todd Chang (Children's Hospital Los Angeles and University of Southern California Keck School of Medicine, West Covina, USA).

## Suppl. Note 2 - Explanation & Elaboration of checklist items

### Title and Abstract - Item 1 (Title)

*Identify the study as an early clinical evaluation, or a similar term, of an application using extended reality (XR), or a more specific term, in the title, including its intended aim.*

– Explanation & Elaboration –

This item focuses on the title of the study and the information it should convey to facilitate the identification and retrieval of early-phase evaluations of applications using extended reality (XR). The item emphasizes the need to clearly state the use of XR and describe the study as an early clinical evaluation or a similar term. It also allows for the inclusion of more specific terms for XR, such as virtual reality or augmented reality, and more specific terms for early clinical evaluation, such as pilot, proof-of-concept, or feasibility study, based on the authors' discretion.

The title of the study plays a crucial role in effectively communicating the key aspects of the research. It should clearly indicate that the study involves the evaluation of an application utilizing XR. By including XR-specific terms like virtual reality or augmented reality in the title, researchers can provide further specificity about the technology employed.

In addition to the use of XR, the title should specify that the study represents an early clinical evaluation or a similar term. This information is essential for literature searches and retrieval, as it helps distinguish early-phase evaluations from other types of studies. Early clinical evaluations often encompass initial feasibility, pilot, or proof-of-concept studies that explore the potential of XR applications in the clinical setting.

If not constrained by word or character limits imposed by journals, authors should consider incorporating other key information in the title. These elements may include:

- Stage of assessment: Although standardized definitions for stages of research on XR applications are currently lacking, referencing frameworks can help specify the stage of assessment. These frameworks may involve categorizations such as early-stage, intermediate-stage, or advanced-stage evaluations.
- Study aim: The title can succinctly summarize the primary objective or research question of the study, providing readers with a clear understanding of the study's purpose.
- Study population: Mentioning the specific target population or patient group being evaluated in the title enhances the relevance and applicability of the study to particular clinical scenarios.
- Study design: If feasible, indicating the study design, such as randomized controlled trial, single-arm study, or observational study, in the title offers insights into the methodology employed.
- (Commercial) name of the application: Including the name of the XR application in the title can aid in identifying other studies that investigate the same application, fostering better collaboration and comparison among researchers.
- Type of evaluation: Briefly stating the type of evaluation, such as efficacy, safety, usability, or feasibility, helps readers grasp the primary focus of the study.

By incorporating these additional elements into the title, researchers can enhance the discoverability and relevance of their early clinical evaluation of XR applications.

### Title and Abstract - Item I (Abstract)

*Provide a (structured) summary of the study.*

*Consider including:*

- *A concise description of the clinical problem/knowledge gap and the rationale for using an application using XR*
- *A concise description of the study methods, including a short description of the application including its name, study population, study setting, main outcomes and assessment methods.*
- *A concise description of the results, including safety and harms outcomes*
- *A short conclusion*
- *If applicable, details about the registration of the study in a publicly available database.*

– Explanation & Elaboration –

This item emphasizes the importance of a clear and sufficiently detailed abstract that accurately reflects the study's conduct and findings. The abstract serves as a crucial entry point for readers, helping them assess the study's quality, relevance, and decide whether to delve into the full article. It should provide a structured summary that includes essential information about the clinical problem, study methods, results, and a conclusion. Additionally, if applicable, information about study registration in a publicly available database should be included.

The abstract is a concise summary of the study and should contain sufficient information to provide a comprehensive understanding of its key aspects within the word count limits set by journals. It plays a crucial role in enabling readers to assess the relevance and quality of the research and aids in retrieving relevant literature from electronic databases.

To ensure transparency and accuracy, the abstract should adhere to the following components:

1. Concise description of the clinical problem/knowledge gap and rationale for using an application using XR: Clearly state the clinical problem or gap in knowledge that the study aims to address. Explain the rationale for utilizing an XR application in addressing the problem, highlighting its potential benefits or advantages over traditional approaches.
2. Concise description of study methods: Provide a brief overview of the study design, including the name of the application being evaluated. Describe the study population, specifying relevant characteristics such as patient demographics or clinical condition. Mention the study setting where the evaluation took place. Briefly outline the main outcomes of interest and the assessment methods employed to measure those outcomes (e.g., validated questionnaires, clinical assessments).
3. Concise description of results, including safety and harms outcomes: Summarize the key findings of the study, focusing on the outcomes of interest. Include relevant data or statistics that support the study's conclusions. If safety and harms outcomes were assessed, provide a summary of those findings to inform readers about the potential risks or adverse effects associated with the XR application.
4. Short conclusion: Present a brief concluding statement that summarizes the main findings of the study and their implications. Highlight any important clinical or research implications that arise from the results.
5. Details about study registration: If the study has been registered in a publicly available database, provide the necessary details such as the name of the database and the registration number. This information helps ensure transparency and facilitates access to additional study details for interested readers.

While word count limitations may restrict the inclusion of all information in the abstract, it is crucial to accurately reflect the contents of the full article. If certain details cannot be included in the abstract, authors should ensure that they are adequately addressed within the main text of the article to provide a comprehensive understanding of the study.

### Introduction - Item 2 (Clinical Problem and Existing Evidence)

*Introduce the clinical problem for which the application using XR was used, including its relevance and a description of (the efficacy of) evidence-based or commonly used interventions or the treatment as usual which is expected to be affected by the application using XR.*

– Explanation & Elaboration –

Item 2 focuses on introducing the clinical problem for which the application using XR is employed. It emphasizes the relevance of the problem and the description of evidence-based or commonly used interventions (standard care) that the XR application is expected to affected (e.g., replaced, amended, improved or optimized). The item highlights the ethical obligation to justify the need for a new trial based on a thorough understanding of the existing scientific literature. It also emphasizes the importance of providing background information on the clinical problem and existing interventions to enable readers to comprehend the necessity of the XR application and facilitate comparison with standard practices.

The introduction of a study should provide a clear and comprehensive understanding of the clinical problem that the XR application aims to address. In line with the Declaration of Helsinki, which emphasizes the ethical responsibility of minimizing risks to research participants, authors should justify the need for a new trial by demonstrating a thorough knowledge of the scientific literature.

To address this item effectively, consider including the following information:

1. Clinical problem and relevance: Describe the specific clinical problem or condition for which the XR application is intended. Explain the relevance of the problem, highlighting its impact on patients, healthcare providers, or the healthcare system. Discuss the challenges, limitations, or gaps associated with current interventions or standard care for this problem.
2. Description of evidence-based or commonly used interventions (standard care): If available, provide a detailed account of the existing evidence-based or commonly used interventions that are currently employed as standard care for the clinical problem. Elaborate on the efficacy, limitations, or drawbacks of these interventions, highlighting any gaps or areas where improvements are needed.
3. Justification for the XR application: Explain why the application of XR is considered a potential solution or improvement over existing interventions. Discuss the unique advantages, potential benefits, or innovative features offered by the XR application. This justification should be grounded in the existing scientific literature, highlighting the rationale for exploring XR as an alternative or complementary approach.
4. Comparison against existing interventions: Emphasize the importance of comparing the XR application against existing standard practices for the same clinical problem. Acknowledge the variations in standard practices across countries or regions, and clarify the specific standard care being compared in the context of the study. This comparison allows readers to understand the potential impact and benefits of the XR application relative to current approaches.

By incorporating these elements into the introduction, authors can provide a clear rationale for the need to evaluate an XR application, demonstrate an understanding of the clinical problem and existing interventions, and justify the ethical basis for conducting the study. This comprehensive introduction enables readers to grasp the significance of the research and the potential impact of the XR application in addressing the clinical problem at hand.

### Introduction - Item 3 (Introduction of the application)

*Introduce the application using XR, including:*

- *Hypotheses for the potential effect; how the application is expected to contribute to the clinical problem.*
- *If available, a concise description of, or a reference to, previous research on the same (or a similar) application.*

– Explanation & Elaboration –

Item 3 focuses on introducing the application using XR in the context of the study. It highlights the importance of stating the hypotheses for the potential effect of the XR application and how it is expected to contribute to addressing the clinical problem. Additionally, if available, the item suggests providing a concise description or reference to previous research on the same or similar application to establish the existing knowledge base and build upon prior work.

When introducing the XR application in the study, consider including the following information:

1. Hypotheses for the potential effect: Clearly state the hypotheses regarding the potential effect of the XR application on the clinical problem. Explain how the application is expected to contribute to addressing the problem, whether it aims to improve patient outcomes, enhance healthcare delivery, optimize treatment efficacy, or provide novel insights. These hypotheses should be grounded in the rationale and evidence supporting the use of XR technology.
2. Description or reference to previous research: If available, provide a concise description of previous research conducted on the same or similar XR application. This description can summarize the main findings, outcomes, or benefits reported in previous studies. Alternatively, referencing relevant literature or citing previous studies that have explored the application's effectiveness or feasibility can demonstrate the existing knowledge base and help build upon prior work. This shows that the current study is not isolated but part of an ongoing effort to investigate the potential of the XR application in addressing the clinical problem.

By including hypotheses for the potential effect and referencing previous research, authors can establish a strong foundation for the study and demonstrate the scientific rationale behind the choice of the XR application. This information enables readers to understand the research context, assess the novelty of the study, and evaluate its potential contributions to the field. Furthermore, building upon previous work helps in establishing a cumulative body of knowledge and encourages collaboration and synthesis of findings in the field of XR applications.

### Introduction - Item II (Objectives)

*Specify the study objectives or hypotheses.*

– Explanation & Elaboration –

Item II focuses on specifying the study objectives or hypotheses. It acknowledges that objectives are the overarching questions that the trial aims to answer, often related to the efficacy of a therapeutic or preventive intervention. Hypotheses, on the other hand, are pre-specified questions being tested to support the objectives and are more specific, allowing for explicit statistical evaluation. It acknowledges that in practice, objectives and hypotheses may overlap and be challenging to differentiate.

When specifying the study objectives or hypotheses, consider the following points:

1. Study objectives: Clearly state the main questions that the trial is designed to answer. These objectives typically revolve around assessing the efficacy, effectiveness, safety, or feasibility of the XR application or intervention being evaluated. Objectives provide a broad perspective on the study's purpose and the outcomes that will be examined.
2. Hypotheses: Define the pre-specified questions being tested within the study. Hypotheses are more specific than objectives and typically focus on comparing groups or assessing the relationship between variables. They are formulated to enable explicit statistical evaluation and hypothesis testing. Hypotheses often address specific aspects of the study, such as the superiority or non-inferiority of the XR application compared to a control group or standard care. Hypotheses help guide the study design, analysis, and interpretation of results.
3. Overlapping nature: Acknowledge that objectives and hypotheses may not always be easily differentiated. In some cases, study objectives may encompass multiple related hypotheses, or the hypotheses themselves may directly align with the overall study objectives. This recognition underscores the interconnectedness and iterative nature of clinical research, where objectives and hypotheses are intertwined and mutually supportive.

By clearly specifying the study objectives or hypotheses, researchers provide a focused and purposeful direction for their investigation. This clarity aids in study design, data analysis, and the interpretation of results. Moreover, explicitly stating the objectives and hypotheses enhances the transparency of the research process and enables other researchers and readers to understand the study's purpose and evaluate its methodology and outcomes effectively.

### Methods and Analysis - Item III (Trial Design and Reporting)

*Provide a reference to ethical approval and, if available, to any (published) study protocol and registration of the study in a publicly available repository.*

– Explanation & Elaboration –

Item III focuses on providing a reference to ethical approval, study protocol, and study registration for research on applications using XR in healthcare. It highlights the importance of obtaining ethics approval and encourages the inclusion of published study protocols and registration in a publicly available repository to enhance transparency and reproducibility. However, it acknowledges that publication or registration may not always be feasible or mandatory for early-phase research.

When addressing Item III, consider the following points:

1. Ethics approval: Ethics approval should be obtained for all research involving applications using XR in healthcare. Ethical approval ensures that the research study meets the necessary ethical standards and safeguards the well-being and rights of study participants.
2. Published study protocol: Although it is highly recommended and desirable, not all early-phase research studies publish their protocols. If a study protocol has been published, provide a reference to the published version. Publishing the study protocol promotes transparency, reproducibility, and allows for a comprehensive understanding of the study design.
3. Registration of the study: Registration of the study in a publicly available repository is beneficial but optional for most early phase evaluations. If the study has been registered, provide the necessary details such as the name of the repository and the registration number. Study registration improves transparency and helps prevent publication bias by ensuring that the research is publicly documented and its findings can be tracked. Registration also allows researchers and readers to access additional details about the study methods and outcomes.
4. Supplementary file for unpublished protocols: If the study protocol was not published or registered, we encourage authors to submit an English version of the study protocol as a supplementary file to the article. This step enhances transparency and reproducibility by providing detailed information about the study design, methods, and data analysis that might not be captured in the main article.

By providing references to ethics approval, published study protocols, and study registration, researchers demonstrate adherence to ethical standards, enhance transparency, and facilitate the reproducibility of study findings. Even when publication or registration is not feasible or mandatory, the inclusion of supplementary files can significantly contribute to the understanding and evaluation of the research methodology and results.

### Methods and Analysis - Item IV (Trial Design and Reporting)

*Describe, and mention the rationale for, the study design. For clarification, it is recommended to use a flow diagram.*

– Explanation & Elaboration –

Item IV emphasizes the importance of describing and providing a rationale for the study design in the research on applications using XR. The item highlights the significance of reproducibility, enabling reader comprehension, and the explanation of why a specific design was chosen to address the main research question.

When addressing Item IV, consider the following points:

1. Describing the study design: Provide a clear and concise description of the study design employed in the research. Explain the overall structure and methodology chosen, such as a randomized controlled trial, a comparative study, a single-arm study, or a qualitative study. Specify the key features of the design, such as the intervention group, control group (if applicable), and any relevant comparison or control conditions.
2. Rationale for the study design: Explain why the chosen study design is appropriate and aligned with the main research question. Justify how the design supports answering the research question effectively, addressing the objectives or hypotheses. Discuss any advantages or strengths of the chosen design, such as its ability to provide causal inference or insights into the application's feasibility, effectiveness, or safety.
3. Flow diagram: Use a flow diagram to visually represent the study design. The flow diagram should include essential elements such as participant recruitment, the timing and duration of using the application, and follow-up assessments, preferably incorporating outcome measures. This diagram provides a clear overview of the study process and helps readers understand the sequence of events and data collection points.
4. Reproducibility and comprehension: By providing a detailed account of the design, researchers enable others to replicate the study or build upon the findings in future research. Additionally, describing the design rationale helps readers understand the reasoning behind the choices made, enhancing comprehension and facilitating critical evaluation of the study's methodology and results.

By describing the study design and providing a rationale, researchers establish a clear framework for their investigation and ensure transparency in the research process. The use of a flow diagram further enhances the visual representation of the design and aids in comprehending the study's structure and procedures. This comprehensive approach improves reproducibility, facilitates reader understanding, and enables effective evaluation of the research methodology and outcomes.

### Methods and analysis - Item 4 (Participants and Setting)

*Describe the setting and locations, including country, where data were collected and processed and where the application using XR was applied and evaluated.*

– Explanation & Elaboration –

Item 4 highlights the significance of describing the setting and locations, including the country, where data collection, processing, and the evaluation of the application using XR took place. It emphasizes the importance of these details for the generalizability and reproducibility of the study findings.

When addressing Item 4, consider the following points:

1. Data collection and processing: Explain the specific locations where data collection and processing activities were conducted, such as hospitals, clinics, or research centers. Emphasize that providing these details helps ensure transparency and reproducibility, allowing other researchers to replicate the study in similar settings and compare their findings. Mentioning the country provides important contextual information about the healthcare system and practices.
2. Application and evaluation: Describe the setting and locations where the application using XR was applied and evaluated, including the country. Highlight that specifying the country adds to the understanding of the research context and enables readers to consider potential variations in healthcare practices, regulations, and technological infrastructure. This information is crucial for assessing the generalizability of the study findings to other similar healthcare settings.
3. Generalizability and reproducibility: Emphasize that providing the setting and country details enhances the generalizability of the study findings. Different countries may have variations in healthcare systems, cultural practices, and patient populations, which can impact the implementation and outcomes of the application using XR. Researchers from other countries or settings can assess the applicability of the study findings to their own contexts based on the reported setting information.

By describing the setting and locations, including the country, researchers enhance the generalizability and reproducibility of their study findings. Transparent reporting allows other researchers to understand the research context and potentially replicate the study in similar settings, contributing to the accumulation of evidence. This information is vital for readers to assess the applicability of the findings to their own healthcare settings and to support the reproducibility of the research in diverse locations.

### Methods and Analysis - Item 5a (Participants and Setting)

*Describe how participants were selected and recruited and provide eligibility criteria.*

– Explanation & Elaboration –

Item 5a focuses on describing how participants were selected and recruited for the study, as well as providing information about the eligibility criteria. It emphasizes the importance of transparency in participant selection and recruitment methods, ensuring that readers can understand the process and assess the representativeness of the study sample.

When addressing Item 5a and providing details about participant selection and recruitment, consider the following points:

1. Selection methods: Explain the purpose and rationale behind the selection methods used in the study. This may include purposeful sampling, convenience sampling, consecutive sampling, or snowball sampling, among others. Clarify why a specific method was chosen and how it aligns with the study objectives or population of interest.
2. Eligibility criteria: Clearly state the eligibility criteria that participants needed to meet in order to be included in the study. This may include factors such as age, gender, specific medical conditions, sensory impairments, contraindications for the application of interest, or cognitive impairments. Providing the eligibility criteria helps readers understand the characteristics of the study population and allows for assessment of the generalizability of the findings to similar populations.
3. Recruitment process: Describe how participants were recruited for the study. This may involve strategies such as advertising, referrals from healthcare professionals, or contacting specific patient groups or organizations. Explain the steps taken to ensure that the recruitment process was transparent, unbiased, and adhered to ethical guidelines. Mention any incentives or compensation provided to participants, if applicable.
4. Participant information and consent: Highlight any measures taken to ensure informed consent and protection of participant rights. This may include providing detailed information about the study, obtaining written consent, and ensuring confidentiality and anonymity of participant data. Emphasize that ethical considerations and regulatory requirements were followed throughout the participant selection and recruitment process.

By providing a clear description of participant selection and recruitment methods, along with the eligibility criteria, researchers enhance the transparency and reliability of their study. Readers can assess the representativeness of the study sample and evaluate the potential impact of participant selection biases on the study findings.

### Methods and Analysis - Item 5b (Participants and Setting)

*Describe who will be applying the application and whether they were trained.*

– Explanation & Elaboration –

Item 5b focuses on providing information about the individuals who will be applying the application using XR in the study, as well as whether they have received training. This item highlights the importance of transparency regarding the qualifications and training of application users to ensure the reliability and validity of the study results.

When addressing Item 5b, consider the following points:

1. Application appliers: Describe the specific individuals or group of professionals who will be responsible for applying the application in the study. This may include healthcare practitioners, researchers, technicians, or other trained personnel. Clearly state their roles and responsibilities in the application process.
2. Qualifications and expertise: Provide information about the qualifications, background, or professional expertise of the application appliers relevant to the specific use of the technology. For example, mention their relevant professional degrees, certifications, or experience in using XR applications. This information helps readers assess the competence and credibility of the application users.
3. Training of application users: Specify whether the individuals who will be applying the application received any training prior to the study.
4. Training assessment: If applicable, mention any assessment or evaluation conducted to ensure the proficiency of application users after training. This could include assessments of their knowledge, practical skills, or adherence to standardized protocols. Reporting on training assessment adds an extra layer of assurance regarding the competency of the application users.

By providing a clear description of who will be applying the application and whether they have received training, researchers enhance the transparency and credibility of their study. Transparent reporting allows readers to evaluate the expertise and competence of the application appliers, which is crucial for ensuring accurate and reliable application administration.

### Methods and Analysis - Item 6 (Intervention and Procedures)

*Provide a description of the application, including its content, hardware, protocol, and set-up, or provide a reference to previous publications where this information is described. Consider supplementing the description with an image, figure or film.*

– Explanation & Elaboration –

Item 6 focuses on providing a comprehensive description of the application being used in the study. This item emphasizes the need to elaborate on various aspects, including the application's content, hardware, protocol, and set-up. The objective is to provide readers with a detailed understanding of the technology and its implementation in the study, potentially supplemented with visual aids like images, figures, or films. Authors may also refer to previous publications where this information has already been described, enhancing reproducibility and enabling readers to access more detailed documentation of the application.

When addressing Item 6, consider the following points:

1. Set-up description: Describe the specific set-up in which the application was used. This includes the physical environment, such as the room or laboratory where the application was employed. If this information has been previously described in other publications, authors can refer to those sources to provide readers with detailed documentation of the set-up.
2. Hardware details: Describe the hardware utilized for the application. Specify the type of immersive technology employed, such as mobile-based, PC-based, console-based, or stand-alone head-mounted display devices. Mention any additional devices used, such as gloves, controls, or (bio)sensors, and provide a rationale for their inclusion. If the hardware details have already been published, authors may refer to those publications to ensure readers have access to comprehensive documentation.
3. Content description: Elaborate on the content of the application. Explain how users interact with the virtual environment and whether they receive feedback. Clarify whether the content is computer-generated (animated), based on 360-degree video or a combination of the latter. Indicate if the content is adaptive or consistent for each user. Mention the senses involved in the application experience. Specify whether the application is single-user or multi-user and if the provider can observe the user's experience. If the content description has been previously published, authors may provide a reference to those publications to ensure readers have access to detailed documentation of the content.
4. Usage details: Describe how the application was used in the study. Explain whether it was self-administered by participants or facilitated by healthcare professionals. If applicable, specify the predefined number of sessions, frequency, and length of sessions. Discuss how participants were instructed in using the application and any additional procedures implemented to mitigate adverse events. Mention any hygiene measures taken and the availability of technical support in case of failure or issues during application usage.

Providing a thorough description of the application, its content, hardware, protocol, and set-up allows readers to gain a comprehensive understanding of the technology and its implementation within the study. This level of detail aids in reproducibility, as other researchers can replicate or adapt the application in similar contexts. Additionally, supplementing the description with visual aids enhances the clarity and visual representation of the application, further aiding readers' comprehension.

### Methods and Analysis - Item 7 (Intervention and Procedures)

*Describe, or provide a reference to, the development process of the application.*

– Explanation & Elaboration –

Item 7 focuses on providing a clear description or reference to the development process of the application used in the study. This information is crucial for understanding the origins, design considerations, and scientific rigor of the application. It allows readers to assess the reliability and validity of the application and aids in reproducibility.

When addressing Item 7, consider the following points:

1. Development process overview: Provide an overview of the development process, highlighting the key steps involved in creating the application. This may include stages such as conceptualization, design, programming, and testing.
2. Development team: Describe the individuals or team involved in the development process. This may include software developers, engineers, designers, clinicians, patients or researchers who contributed to the creation of the application. Highlight their expertise and roles within the team.
3. Content determination: Explain how the content of the application was determined. This may involve referencing theoretical frameworks, scientific literature, or clinical guidelines that informed the content development.
4. Conceptual, theoretical, and scientific framework: Discuss the conceptual, theoretical, and scientific framework that guided the development of the application. Explain the underlying principles or theories that shaped its design and functionality.
5. Prototypes and testing: Mention if prototypes were created during the development process and if and by whom these prototypes were tested. Highlight any important changes or improvements made based on user feedback or testing results.
6. Current study version: Specify the version of the application used in the current study. This allows readers to understand the specific iteration of the application and provides a reference point for future studies using different versions.
7. Software used: Indicate the software tools or platforms used for developing and running the application. This includes programming languages, development frameworks, or specialized software packages. Provide details about the software versions used, if applicable.
8. Reference to previous publications: If the development process has been previously described in detail in another publication, provide a reference to that publication. This allows readers to access more comprehensive information about the development process, especially when space limitations in the current article prevent a detailed description.

By providing a clear account or reference to the development process, authors ensure transparency, enhance the reproducibility of the study, and enable readers to evaluate the quality and scientific basis of the application used in the research.

### Methods and Analysis - Item 8 (Intervention and Procedures)

*Describe the participant timeline in sufficient detail to allow replication, including all procedures, co-interventions (if applicable), and (follow-up) assessments.*

– Explanation & Elaboration –

Item 8 focuses on providing a comprehensive participant timeline that outlines the sequence of procedures, co-interventions (if applicable), and assessments conducted throughout the study. This information is vital for replication purposes as it allows other researchers to follow the study's protocol and procedures accurately. By providing a detailed participant timeline, authors enhance transparency, facilitate the evaluation of study validity, and enable the reproducibility of the research.

When addressing Item 8, consider the following points:

1. Participant flow: Present a visual or textual representation of the participant flow throughout the study, including the number of participants at each stage (e.g., screening, enrollment, intervention, follow-up, analysis). This flowchart or description should clearly indicate the progression of participants and any deviations or dropouts encountered during the study.
2. Procedures: Describe in detail all procedures conducted with participants, including the specific actions, interventions, or assessments performed at each stage. This may include baseline assessments, randomization procedures, administration of the intervention (e.g., XR application), follow-up assessments, and any additional procedures conducted during the study.
3. Co-interventions (if applicable): If co-interventions were used alongside the XR application, describe them in detail. This includes any concurrent treatments, therapies, or interventions that participants received alongside the primary intervention. Specify the type, frequency, duration, and rationale for using co-interventions.
4. Follow-up assessments: Outline the specific assessments conducted during follow-up periods. This includes the timing, frequency, and specific measures employed to evaluate participant outcomes or responses over time. If different assessments were conducted at different time points, clearly indicate the differences and provide a rationale for their inclusion.
5. Timing and duration: Provide a clear timeline indicating the duration of each study phase, including the intervention period and any follow-up periods. Specify the start and end dates or durations for each phase to allow for accurate replication and understanding of the study timeline.
6. Data collection points: Clearly indicate the time points or intervals at which data were collected from participants. This ensures that readers can accurately interpret the temporal aspects of the study and understand the intervals between assessments or interventions.
7. Study milestones: Highlight any significant study milestones or events that occurred during the participant timeline. This may include interim analyses, protocol modifications, safety reviews, or other critical time points during the study.
8. Assessments and outcome measures: Specify the specific assessments and outcome measures employed at each stage of the participant timeline. This includes validated instruments, questionnaires, clinical assessments, or other measurements used to evaluate participant outcomes, adherence, or safety.
9. Data management and monitoring: Describe any procedures implemented to ensure proper data management and monitoring throughout the participant timeline. This may include data quality checks, data entry procedures, monitoring of adverse events, or data oversight measures.

By providing a detailed participant timeline, authors enable other researchers to replicate the study accurately and evaluate the validity and reliability of the research. The comprehensive description of procedures, co-interventions, and assessments enhances transparency and ensures that the study can be reproduced with fidelity.

### Methods and Analysis - Item V (Intervention and Procedures)

*Describe and give a rationale for the control conditions or provide a rationale for not using one.*

– Explanation & Elaboration –

Item V focuses on the importance of describing the control conditions in a study or providing a rationale for not including a control group. This item emphasizes the need to clearly explain the purpose and design of the control conditions, which serve as a reference point for evaluating the effectiveness and safety of the application being studied. Additionally, if a placebo was used, authors should provide a description of its development to enhance transparency and facilitate the understanding of placebo.

When addressing Item V, consider the following points:

1. Control condition description: Provide a detailed description of the control condition(s) used in the study. This includes explaining the nature of the control condition(s), such as standard care, no intervention, waitlist control, active comparator, or sham procedure. Describe the rationale for choosing a particular control condition and how it relates to the research question or objective of the study.
2. Rationale for control conditions: Clearly articulate the reasons for including control conditions or for not using one. If a control group is included, discuss the need for a reference point to evaluate the effectiveness, safety, or other outcomes of the intervention. Explain how the control condition(s) helps address potential confounding factors, placebo effects, or biases that could influence the study results.
3. Placebo development (if applicable): If a placebo was used as part of the control condition, describe its development process. Explain how the placebo was designed to mimic the intervention in appearance, administration, or other relevant aspects. Detail any considerations made to ensure the placebo's inertness or lack of specific active ingredients or effects. This description enhances transparency and enables readers to understand the efforts made to create a suitable placebo for the study.
4. Blinding procedures: If blinding (i.e., masking) was employed, explain the methods used to ensure blinding of participants, researchers, and/or assessors to the intervention and control conditions. Discuss the rationale for blinding and its potential impact on reducing biases and enhancing the internal validity of the study.
5. Rationale for not using a control condition: If a control group was not included in the study design, provide a detailed rationale for this decision. Explain why a control condition was deemed unnecessary or ethically inappropriate based on the specific context, research question, or practical considerations. Discuss potential limitations or biases associated with the absence of a control group and how they were mitigated or addressed in the study design.
6. Comparability of control conditions: If multiple control conditions were used, compare and discuss their similarities and differences. Explain how each control condition addresses specific aspects of the research question or objective and provide a rationale for their inclusion.
7. Pilot studies or previous research: If applicable, refer to any pilot studies or previous research that informed the selection or development of the control conditions. Discuss how the findings from these preliminary studies influenced the decision-making process and contributed to the rationale for the control conditions employed in the current study.

By describing the control conditions or providing a rationale for not using one, authors enhance the transparency and validity of their study. Clear explanations of the control conditions help readers understand the study design and the basis for evaluating the intervention's effectiveness. Additionally, providing a detailed description of the placebo development, if applicable, ensures transparency and enables readers to evaluate the suitability and inertness of the placebo used.

### Methods and Analysis - Item VI (Outcomes)

*Describe all pre-specified primary and secondary outcomes, including how and when assessed.*

– Explanation & Elaboration –

Item VI focuses on the need to describe all pre-specified primary and secondary outcomes in a study, including the methods and timing of assessment. This item emphasizes the importance of transparently reporting the specific outcomes measured and the instruments used for quantitative measures, as well as the methods and assessors involved in qualitative measures. It further highlights the significance of providing information on the psychometric quality of quantitative instruments, if available, to assess their reliability and validity.

When addressing Item VI, consider the following points:

1. Pre-specified primary and secondary outcomes: Clearly describe all primary and secondary outcomes that were predetermined in the study protocol or research plan. These outcomes represent the specific variables or constructs that the study aims to measure to evaluate the intervention's effectiveness or other study objectives. Provide a concise and comprehensive list of these outcomes.
2. Quantitative outcome measures: For quantitative measures, provide a detailed description of the instruments used to assess the outcomes. Include information about the instrument's name, purpose, and any specific domains or constructs it measures. If available, discuss the psychometric quality of the instrument, including its reliability and validity. This information helps readers evaluate the robustness of the quantitative measures used.
3. Timing of outcome assessment: Specify the time points at which the outcomes were assessed (e.g., baseline, post-intervention, follow-up). Describe the rationale for the chosen timing, considering factors such as the expected duration of intervention effects or the desired evaluation time points for specific outcomes.
4. Qualitative outcome measures: For qualitative measures, explain the methods employed to assess the outcomes. Specify whether focus groups, interviews, or other qualitative research techniques were used. Describe the duration of interviews or focus groups and any specific interview or focus group guides or protocols used. Additionally, provide information about the occupation, experience, and training of the assessors conducting the qualitative assessments.
5. Mixed methods approaches: If both quantitative and qualitative measures were used, explain how they were integrated to assess the outcomes. Describe how the data from different methods were combined or analyzed to provide a comprehensive understanding of the outcomes.
6. Assessment tools and guidelines: If standardized tools or guidelines were used to assess outcomes, provide references or citations to these tools and briefly describe their purpose and relevance to the study.
7. Pre-specification of outcomes: Discuss whether the primary and secondary outcomes were pre-specified in the study protocol or research plan before data collection began. Pre-specification helps minimize outcome reporting bias and enhances the study's transparency and credibility.
8. Assessors' training and calibration: If relevant, provide information on any training or calibration procedures implemented for assessors to ensure consistency and reliability in outcome assessment. Describe any efforts made to minimize inter-rater variability or bias among assessors.
9. Assessing multiple dimensions or domains: If the outcomes encompass multiple dimensions or domains, clearly describe each dimension or domain and the specific measures used for each. This allows readers to understand the breadth and depth of the outcomes assessed.

By describing all pre-specified primary and secondary outcomes, as well as the methods and timing of assessment, authors enhance the transparency and replicability of their study. Clearly reporting the instruments used for quantitative measures, including their psychometric quality, enables readers to evaluate the reliability and validity of the data collected. Similarly, providing details about the methods and assessors involved in qualitative measures ensures transparency and allows readers to assess the rigor of the qualitative assessments.

### Methods and Analysis - Item 9 (Outcomes)

*Describe how safety and harm outcomes were assessed. Describe which, and how, other XR-specific outcomes were assessed, such as performance, usability, presence, perspectives, and acceptability.*

– Explanation & Elaboration –

Item 9 focuses on the assessment of safety and harm outcomes, as well as the assessment of other XR-specific outcomes related to performance, usability, presence, perspectives, and acceptability. This item highlights the importance of transparently reporting the methods and measures used to assess these outcomes, emphasizing the need to address safety and harms in all early-phase XR studies. Additionally, it suggests specific questionnaires that can be utilized for assessing presence and cybersickness in XR studies.

When addressing Item 9, consider the following points:

1. Safety and harm outcomes assessment: Assessing safety and harms is essential in early-phase evaluations of applications using XR. Describe the methods used to assess safety and harm outcomes. This may include monitoring and reporting adverse events or any negative effects experienced by participants during or after XR exposure. Safety and harm outcomes should always be addressed in XR studies to ensure participant well-being and minimize risks. For first-in-human studies, the Simulator Sickness Questionnaire (SSQ) is recommended for assessing adverse effects related to simulator sickness.
2. XR-specific outcomes assessment: If applicable, describe the specific XR-related outcomes assessed in the study. This may include the following:
   1. Effects (psychological, physiological, physical): Explain how psychological, physiological, or physical effects were measured or evaluated in relation to the XR application.
   2. Performance: Describe the methods used to assess performance outcomes, such as error rates or task completion time. Provide details on the specific performance measures employed and how they were collected.
   3. Usability: Describe the methods used to assess the usability of the XR application. This may include user experience evaluations, task success rates, or qualitative feedback from participants regarding the ease of use and navigation of the XR application.
   4. Presence: Explain how presence was assessed in the XR study. The Igroup Presence Questionnaire (IPQ) is recommended for the assessment of presence.
   5. Interruptions or malfunctions: If applicable, describe how interruptions or malfunctions of the XR intervention were assessed or recorded. For example, report the number of sessions interrupted due to technical issues or malfunctions.
   6. Acceptability: Explain how the acceptability of the XR intervention was assessed, considering both the perspectives of end-users and those offering the application. This may involve surveys, interviews, or qualitative feedback to gauge satisfaction, ease of use, engagement, and willingness to recommend the XR application.
   7. Perspectives: Describe the methods used to assess healthcare professionals’ and participants' perspectives on the XR intervention. This may include satisfaction ratings, perceived ease of use, engagement levels, or willingness to recommend the XR application to others.

By transparently describing the methods and measures used to assess safety and harm outcomes, as well as other XR-specific outcomes, authors enhance the transparency and reliability of their study. The emphasis on addressing safety and harms underscores the importance of participant well-being in XR research. Additionally, the recommendation of specific questionnaires, such as the Simulator Sickness Questionnaire and the Igroup Presence Questionnaire, provides guidance on validated measures for assessing adverse effects and presence in XR studies.

### Methods and Analysis - Item VII (Sample Size)

*Provide a justification for the sample size.*

– Explanation & Elaboration –

Item VII highlights the importance of justifying the chosen sample size in a study. This item emphasizes the need to provide a rationale for the sample size, whether it was determined through a formal sample size calculation or estimated based on presumptions. Authors are encouraged to describe how they justified the expected effect or prior probability distribution when using a formal sample size calculation. In cases where no predetermined sample size was used, authors should explain how they determined that the sample size used in the study was sufficient.

When addressing Item VII, consider the following points:

1. Sample size determination: Explain the process by which the sample size was determined for the study. If a formal sample size calculation was conducted, describe the method used (e.g., power analysis) and provide details on the expected effect size, significance level, power, or other relevant parameters used in the calculation. Justify the chosen effect size or prior probability distribution based on existing literature, pilot studies, or expert opinions. This demonstrates the scientific basis for the sample size determination.
2. Estimated guess: If the sample size was estimated based on an educated guess rather than a formal calculation, provide the presumptions or considerations that influenced the choice. These may include practical constraints, available resources, or previous studies with similar objectives. Explain why the estimated sample size was considered appropriate for achieving the study's objectives and providing meaningful results.
3. Sufficiency of the sample size: In cases where no predetermined sample size was used and the sample size was determined during the study, describe the process by which it was determined that the sample size used was sufficient. This could include reaching a point of saturation in qualitative research, achieving statistical significance in quantitative analyses, or using interim analyses to determine the adequacy of the sample size. Clarify the rationale behind considering the obtained sample size as adequate for drawing valid conclusions from the study.

Providing a justification for the sample size enhances the transparency and validity of the study. By describing the method used to determine the sample size or the considerations behind an estimated guess, authors demonstrate the thought process and scientific rigor applied in planning the study. This information allows readers to evaluate the reliability and generalizability of the study results based on the sample size justification.

### Methods and Analysis - Item VIII (Analysis)

*Provide a detailed description how primary and secondary outcomes were analyzed, including any pre-specified comparisons or stratifications.*

– Explanation & Elaboration –

Item VIII emphasizes the need to provide a comprehensive description of how the primary and secondary outcomes were analyzed in the study. This item highlights the importance of transparency regarding the analytical methods, including any pre-specified comparisons or stratifications. Additionally, for mixed methods studies, authors are encouraged to describe how data integration was performed, while for qualitative studies, the approach used should be specified.

When addressing Item VIII, consider the following points:

1. Analytical methods: Describe the specific analytical methods used to analyze the primary and secondary outcomes. This may include statistical tests, modeling approaches, or qualitative analysis techniques. Provide sufficient details to allow readers to understand the methods employed.
2. Pre-specified comparisons or stratifications: If any pre-specified comparisons or stratifications were planned, explain them in detail. These may include subgroup analyses, comparisons between treatment arms, or analyses based on specific variables. Justify the chosen comparisons or stratifications based on the research objectives, existing literature, or clinical relevance.
3. Mixed methods studies: For studies that employed mixed methods, describe how data integration was performed. Explain the process of combining quantitative and qualitative data, including any specific frameworks or methods used. Elaborate on how the different types of data were integrated, whether through sequential or concurrent data analysis, and provide details on any steps taken to ensure the compatibility and coherence of the integrated findings.
4. Qualitative studies: If the study utilized qualitative methods, specify the approach used for data analysis. Common approaches include thematic analysis, grounded theory, phenomenology, or content analysis. Describe the steps involved in the analysis process, such as coding, categorization, and interpretation. Provide information on any software or analytical tools used, and mention any measures taken to ensure rigor and trustworthiness of the qualitative analysis.

By providing a detailed description of how the primary and secondary outcomes were analyzed, authors enhance the transparency and reproducibility of their study. Clear reporting of the analytical methods and any pre-specified comparisons or stratifications allows readers to evaluate the appropriateness and validity of the data analysis. Additionally, for mixed methods and qualitative studies, describing the data integration process and the approach to qualitative analysis further enhances the understanding and trustworthiness of the study findings.

### Methods and Analysis - Item IX (Protocol alterations)

*Describe changes to the methods or protocol, including to procedures, study outcomes, eligibility criteria and analysis plan, after study commencement, with reasons and, if applicable, report whether the study registration was updated.*

– Explanation & Elaboration –

Item IX emphasizes the importance of reporting any changes made to the study methods or protocol after the study has commenced. This item highlights the need for transparency and provides an opportunity for authors to explain the reasons behind the changes. Additionally, authors should indicate whether the study registration, such as a clinical trial registration, was updated to reflect these modifications.

When addressing Item IX, consider the following points:

1. Changes to methods or protocol: Describe any modifications made to the study methods or protocol after the study has started. This may include changes to procedures, study outcomes, eligibility criteria, or the analysis plan. Clearly explain the nature of each change and provide sufficient details for readers to understand the implications.
2. Reasons for changes: Provide a rationale for each change that was implemented. Explain the factors or considerations that led to the modification. This could include new information or evidence that emerged during the study, logistical challenges, unexpected events, or feedback from participants or stakeholders. Justify each change based on its impact on the study design, scientific validity, or feasibility.
3. Study registration update: If the study was registered in a registry or database, such as a clinical trial registration, indicate whether the registration was updated to reflect the changes. If an update was made, provide the necessary details, such as the date of the update and any registration numbers or identifiers associated with the study. This ensures transparency and helps readers access the most up-to-date information about the study.

By describing changes to the methods or protocol, providing reasons for the modifications, and reporting updates to study registration, authors enhance the transparency and integrity of their research. Transparent reporting allows readers to understand the evolution of the study and evaluate the potential impact of the changes on the study outcomes and conclusions. Updating the study registration, when applicable, ensures that the registered information aligns with the actual conduct of the study, reducing any discrepancies between planned and implemented methods.

### Results - Item X (Participants Flow and Recruitment)

*Describe the timeframe of recruitment and follow-up and the participant flow, including number of patients screened and included, receiving the intervention and being included in each analysis. Report if, and why, the study was prematurely terminated. The use of a flow diagram is highly recommended.*

– Explanation & Elaboration –

Item X focuses on providing a comprehensive description of the study's participant flow, including the timeline of recruitment and follow-up, reasons for study termination, and key numbers related to participant screening, eligibility, participation, and analysis. The inclusion of a flow diagram is strongly recommended as it visually presents the participant flow and enhances the transparency of the study.

When addressing Item X, consider the following points:

1. Timeframe of recruitment and follow-up: Describe the duration of the recruitment phase, including the start and end dates, as well as any specific time points or intervals for follow-up assessments. Provide a clear timeline that reflects the study's enrollment and data collection periods. Justify the chosen timeframe based on study objectives, feasibility, and logistical considerations.
2. Reasons for study termination or end: If applicable, explain why the study was stopped or ended before reaching the planned completion date. This may include reasons such as meeting predefined stopping criteria, challenges with recruitment, funding limitations, ethical considerations, or unforeseen events. Justify the decision to end the study and describe any actions taken to minimize bias or mitigate potential implications.
3. Participant flow and numbers: Provide a detailed participant flow diagram that visually illustrates the progression of participants through the study. Include the number of patients screened, the number found eligible and approached, the number not meeting eligibility criteria along with reasons, the number declining participation along with reasons, the total number of inclusions, and the number of patients who received or did not receive the intervention along with reasons. Additionally, report any losses or exclusions after inclusion, including reasons for dropouts or withdrawals from the study.
4. Flow diagram: Include a flow diagram that visually represents the participant flow. The flow diagram should clearly depict the number of participants at each stage of the study, starting from the screening phase to the final analysis. The diagram should be designed according to the Consolidated Standards of Reporting Trials (CONSORT) guidelines or other relevant reporting standards.

By providing a detailed description of the timeframe of recruitment and follow-up, reasons for study termination, and participant flow with relevant numbers, authors enhance the transparency and reproducibility of their research. The inclusion of a flow diagram simplifies the understanding of participant progression and facilitates the assessment of study quality and potential sources of bias. Clear reporting of participant numbers and reasons for exclusion or withdrawal aids in the interpretation and generalizability of study findings.

### Results - Item XI (Baseline Data)

*Describe, or add a table depicting, baseline and treatment-related characteristics. If applicable, describe and specify any concurrent measures.*

– Explanation & Elaboration –

Item XI focuses on providing a comprehensive description of baseline and treatment-related characteristics of participants in the study. Additionally, if applicable, authors should describe and specify any concurrent measures used. These details are important for understanding the participant population, assessing the comparability of groups, and evaluating the impact of the intervention.

When addressing Item XI, consider the following points:

1. Baseline characteristics: Provide a detailed description of baseline characteristics that are relevant to the study. This may include demographic information such as age and gender, comorbidities, and any other pertinent baseline factors. Consider including information on participants' familiarity with XR or gaming, as well as their expectations about XR and motivations for participation. These additional factors help contextualize the study population and potential influences on their experiences.
2. Treatment-related characteristics: Describe the treatment-related characteristics of participants, focusing on factors that may impact the study outcomes. This could include the type of disease or condition being treated, the stage or severity of the disease, and the duration of admission to the hospital or healthcare setting. These details provide important context for interpreting the results and understanding the implications of the intervention.
3. Concurrent measures: If applicable, describe and specify any concurrent measures used in the study. This may include psychophysiological measures, retrospective measures such as questionnaires, or any other measurements collected alongside the primary outcomes. Provide a brief description of these measures, their purpose, and their relevance to the study objectives. Consider including information on the psychometric properties or validation of the measures if available.
4. Table depiction: Consider presenting the baseline and treatment-related characteristics in a table format. This allows for a clear and concise presentation of the data, making it easier for readers to grasp the key information at a glance. Ensure that the table includes all relevant variables and provides sufficient detail to capture the diversity of the study population.

By describing baseline and treatment-related characteristics, as well as any concurrent measures, authors enhance the transparency and comprehensiveness of their study. Including information on familiarity with XR or gaming, expectations about XR, motivations for participation, and baseline characteristics such as age, gender, and comorbidities provides valuable insights into the participant population. Reporting treatment-related characteristics and concurrent measures helps readers understand the context and potential influences on the study outcomes.

### Results - Item XII (Main Results)

*Report on all pre-specified outcomes that are available. Consider using tables, figures and/or graphs to illustrate results.*

– Explanation & Elaboration –

Item XII focuses on reporting all pre-specified outcomes that are available in the study, including any comparisons between pre-specified subgroups if conducted. The objective is to provide a comprehensive presentation of the study results and ensure transparency in reporting. Visual aids such as tables, figures, and graphs can enhance the clarity and accessibility of the results.

When addressing Item XII, consider the following points:

1. Pre-specified outcomes: Report on all pre-specified outcomes that were determined in advance and included in the study protocol or analysis plan. Provide a clear and concise description of each outcome measure. This includes both primary and secondary outcomes.
2. Comparison between pre-specified subgroups: If pre-specified subgroup analyses were conducted, report on the outcomes for each subgroup and any comparisons made between them.
3. Use of visual aids: Consider using tables, figures, and/or graphs to present the results in a visually appealing and informative manner. Tables can be used to summarize the data for each outcome, including relevant statistical measures such as means, standard deviations, or effect sizes. Figures and graphs can be employed to illustrate the patterns or trends in the data, making it easier for readers to interpret the results. Ensure that the visual aids are clearly labeled, properly formatted, and accompanied by appropriate captions or legends.

By reporting all pre-specified outcomes, including comparisons between pre-specified subgroups if conducted, authors ensure transparency and completeness in reporting the study results. The use of visual aids such as tables, figures, and graphs enhances the accessibility and interpretation of the data, facilitating a clear understanding of the findings.

### Results - Item 10 (Extended Reality and Human Factors)

*Include information about the usage of the application, such as duration, frequency, number of sessions, error rates and number of sessions requiring interruption or discontinuation including reasons.*

– Explanation & Elaboration –

Item 10 emphasizes the importance of providing detailed information about the usage of the application in the study. This includes various aspects such as the duration of each session, the frequency of usage, the number of sessions completed by participants, error rates, and instances where sessions required interruption or discontinuation. Reporting these usage parameters is crucial for understanding how the application was implemented and any challenges encountered during its usage.

When addressing Item 10, consider the following points:

1. Duration of each session: Describe the average or typical duration of each application session completed by the participants. Include information on whether the session duration was standardized or varied among participants.
2. Frequency of usage: Specify the frequency at which participants were instructed or expected to use the application. This could be daily, weekly, or based on any other predetermined schedule. If participants had flexibility in determining the usage frequency, describe the range or distribution of usage frequencies observed in the study.
3. Number of sessions completed: Report the total number of application sessions completed by each participant. Provide information on any criteria or guidelines that determined the recommended or expected number of sessions. If there were variations in the number of sessions completed among participants, explain the reasons for these differences.
4. Error rates: Describe the occurrence of errors or technical issues encountered during the usage of the application. This may include software glitches, hardware malfunctions, or any other challenges that affected the smooth operation of the application. Quantify the error rates or provide a qualitative description of the types and severity of errors encountered.
5. Interruptions or discontinuations: Report the number of application sessions that required interruption or discontinuation and provide the reasons behind these interruptions. Common reasons may include participant discomfort, technical difficulties, adverse events, or other unforeseen circumstances. Clearly differentiate between sessions that were interrupted temporarily and resumed later versus sessions that were permanently discontinued.

Including information about the usage of the application provides valuable insights into the practical implementation of the intervention. It helps readers understand the intensity and feasibility of application usage within the study context. Additionally, reporting error rates, interruptions, and discontinuations highlights the challenges and limitations encountered during the usage of the application, contributing to a more comprehensive evaluation of the intervention's effectiveness.

### Results - Item 11 (Extended Reality and Human Factors)

*If assessed, report on XR-specific outcomes, such as performance, usability, presence, perspectives, and acceptability.*

– Explanation & Elaboration –

Item 11 emphasizes the need to report on specific outcomes related to XR (XR) technology in the study. These outcomes go beyond traditional measures and focus on the unique aspects of XR applications, including performance, usability, presence, perspectives, and acceptability. Reporting these XR-specific outcomes provides valuable insights into the effectiveness and user experience of the application.

When addressing Item 11, consider the following points:

1. Performance: Quantify and report the performance outcomes, highlighting any significant findings or patterns observed. This could involve measuring objective performance indicators, such as completion time, accuracy, task efficiency, or any other relevant metrics.
2. Usability: Report the results of the usability assessment, including any areas of improvement or strengths identified.
3. Presence: Report the presence outcomes, including the average scores or any significant differences observed between subgroups or conditions.
4. Perspectives: Summarize the perspectives expressed by participants, highlighting common themes or notable insights that emerged from their feedback.
5. Acceptability: Report on the acceptability of the XR application , including any concerns, challenges, or positive feedback received, among participants and other stakeholders involved in the study.

By reporting on extended-reality specific outcomes, researchers gain a deeper understanding of the impact and user experience of the XR application. These outcomes provide insights into the effectiveness, usability, presence, perspectives, and acceptability of the intervention, enhancing the overall evaluation of the study findings. It is important to consider the guidelines and recommendations provided in the explanation and elaboration text for Item 9, which highlights the specific XR-related outcomes and questionnaires that are recommended for assessment.

### Results - Item 12 (Safety and Harms)

*Report on safety and harms, including unintended effects, both during and after using the application.*

– Explanation & Elaboration –

Item 12 emphasizes the importance of reporting on the safety and potential harms associated with using the application, including unintended effects. This includes reporting any short-term and long-term unintended effects, whether physical or physiological in nature. Understanding the safety profile of the intervention is crucial for evaluating its overall risk-benefit balance.

When addressing Item 12, consider the following points:

1. Harms and unintended effects: Report on any observed harms or unintended effects that occurred during or after using the application. This includes both short-term effects that may have occurred immediately or shortly after application usage, as well as any long-term effects that persisted beyond the immediate intervention period. Document and describe any physical or physiological unintended effects, such as discomfort, dizziness, headache, nausea, changes in heart rate, or other relevant outcomes.
2. Severity and duration: Evaluate and report the severity and duration of the observed harms and unintended effects. This can include categorizing the severity of adverse events using a standardized scale or reporting the duration of any observed symptoms or physiological changes. Provide a clear description of the nature and magnitude of the identified effects, as well as any potential impact on participants' well-being or daily functioning.

By reporting on safety and harms, including short- and long-term unintended effects, both physical and physiological, researchers provide crucial information regarding the safety profile of the application. This transparency allows readers and stakeholders to evaluate the potential risks and benefits associated with using the application. It is important to assess and report any observed unintended effects comprehensively to ensure the accuracy and completeness of the safety assessment.

### Discussion and Conclusion - Item 13 (Generalizability and Impact)

*Discuss (potential) impact of study findings and generalizability, including barriers for the use/implementation of the application.*

– Explanation & Elaboration –

Item 13 highlights the importance of discussing the potential impact of the study findings and their generalizability. This item also emphasizes considering the conservative nature of impact assessment, particularly in early-phase clinical evaluations. Additionally, if XR-specific outcomes were assessed, it is crucial to include their discussion within the broader context of study findings and generalizability.

When addressing Item 13, consider the following points:

1. Impact of study findings: Discuss the potential impact of the study findings in the context of the application's target population and the specific clinical or research area being addressed. Consider the implications of the findings on patient outcomes, healthcare practices, or research methodologies. Emphasize the conservative nature of impact assessment, given the early-phase clinical evaluation, and highlight any limitations or uncertainties that may affect the generalizability of the findings.
2. Generalizability: Reflect on the generalizability of the study findings beyond the specific study population and setting. Discuss the potential applicability of the application in other similar contexts, patient populations, or healthcare systems. Consider factors that may influence the generalizability, such as variations in patient characteristics, clinical practices, resource availability, or technological infrastructure.
3. Barriers for use/implementation: Identify and discuss any barriers or challenges that may hinder the use or implementation of the application in real-world settings. This may include factors such as cost, technical requirements, training needs, workflow integration, regulatory considerations, or acceptance by end-users or healthcare providers. Provide a thoughtful analysis of these barriers and potential strategies for addressing them.
4. XR-specific outcomes: If XR-specific outcomes were assessed, discuss their implications within the broader context of the study findings and generalizability. Consider the impact of performance, usability, presence, perspectives, and acceptability outcomes on the overall evaluation of the application. Discuss the potential benefits, limitations, and considerations specific to the XR technology used in the study.

By discussing the (potential) impact of study findings and generalizability, as well as barriers for the use/implementation of the application, researchers provide valuable insights into the practical implications of their work. Emphasizing the conservative nature of impact assessment acknowledges the early-phase clinical evaluation and promotes responsible interpretation of the study findings. Incorporating the discussion of XR-specific outcomes, if assessed, enhances the comprehensive understanding of the application's potential benefits and challenges in real-world settings.

### Discussion and Conclusion - Item 14 (Safety and Harms)

*Discuss safety and instances of harm, including their possible effects on study findings, implications for future use of the applications and whether they can prevented or mitigated.*

– Explanation & Elaboration –

Item 14 emphasizes the importance of discussing safety and instances of harm related to the use of the application. This item highlights the need to consider the potential effects of safety issues on study findings, as well as the implications for the future use of the application. It also prompts researchers to explore preventive or mitigating measures that can be implemented to enhance the safety of the application.

When addressing Item 14, consider the following points:

1. Safety and instances of harm: Discuss any safety concerns or instances of harm that were observed during the study. This may include physical, physiological, or psychological effects experienced by participants while using or after having used the application. Provide a detailed description of the nature and severity of these safety issues or harms, ensuring transparency in reporting. Consider short-term and long-term unintended effects and their implications.
2. Effects on study findings: Reflect on the possible effects of safety issues or instances of harm on the study findings. Discuss whether the presence of these issues could have influenced the outcome measures, participant adherence, or data quality. Address the potential limitations or biases that may have arisen due to safety-related factors and their impact on the interpretation and generalizability of the study results.
3. Implications for future use: Discuss the implications of safety issues or instances of harm for the future use of the application. Consider whether the observed safety concerns pose significant barriers or limitations for wider implementation or adoption of the technology. Explore the potential impact on patient outcomes, user acceptance, or healthcare practices. Discuss whether modifications, additional training, or further research are necessary to address the identified safety concerns.
4. Prevention and mitigation: Address whether the identified safety issues could have been prevented or mitigated. Discuss any measures taken during the study to minimize the risk of harm and whether they were effective. Explore potential strategies or recommendations for preventing or mitigating similar safety issues in future applications or research studies. Consider the role of user training, monitoring protocols, technical improvements, or modifications to the intervention design.

By discussing safety and instances of harm, researchers demonstrate a commitment to transparency and participant well-being. Considering the possible effects of safety issues on study findings enhances the interpretation and validity of the results. Exploring the implications for future use and discussing preventive or mitigating measures promotes the responsible development and implementation of applications, ensuring the safety of users and optimizing the potential benefits of the technology.

### Discussion and Conclusion - Item 15 (Ethics)

*Describe ethical considerations, including benefits and risks, for the current and future use of the application.*

– Explanation & Elaboration –

Item 15 highlights the importance of discussing ethical considerations related to the current and future use of the application. This item emphasizes the need to address both the potential benefits and risks associated with the technology, considering the specific context of early-phase evaluation of XR applications in healthcare.

When addressing Item 15, consider the following points:

1. Potential benefits: Discuss the anticipated benefits of using the application in healthcare, both for participants in the current study and for future users. This may include improvements in patient outcomes, enhanced therapeutic interventions, increased accessibility to care, or advancements in healthcare practices. Consider the potential societal benefits and the potential to address unmet needs in healthcare.
2. Potential risks: Identify and discuss the potential risks associated with the use of the application. These risks may include physical risks, such as simulator sickness or discomfort, psychological risks, such as adverse psychological reactions or increased anxiety, or social risks, such as stigmatization or privacy breaches. Consider the specific risks that may arise due to the immersive and interactive nature of XR applications.
3. Equity and access: Address any ethical considerations related to equity and access to the technology. Discuss the potential for disparities in access to XR applications and the importance of ensuring equitable distribution of benefits. Consider the potential impact on vulnerable populations and any efforts made to address these considerations.

Examples of ethical considerations specific to early-phase evaluation of XR applications in healthcare may include:

- Ensuring participant safety during the use of immersive technologies, such as monitoring for adverse effects and providing immediate support or interventions when needed.
- Safeguarding the rights and well-being of vulnerable populations, such as children, individuals with cognitive impairments, or patients with severe health conditions.
- Considering the potential ethical implications of using XR technologies in sensitive or emotionally challenging contexts, such as therapy for trauma or end-of-life care.

By addressing ethical considerations, researchers demonstrate a commitment to responsible and ethical practices, safeguarding the well-being and rights of end-users. Considering both the potential benefits and risks of XR applications promotes ethical decision-making and ensures that the technology is implemented in a manner that aligns with ethical principles and societal values.

### Discussion and Conclusion - Item XIII (Strengths and Limitations)

*Discuss study strengths and limitations, including sources of potential bias.*

– Explanation & Elaboration –

Item XIII focuses on discussing the strengths and limitations of the study, including potential sources of bias. This item highlights the importance of critically evaluating the study's design, methodology, and data analysis to provide a balanced assessment of its strengths and weaknesses. Additionally, it emphasizes the need to identify and address potential biases that may have influenced the study's outcomes. By acknowledging both the strengths and limitations of the study, researchers can provide a more comprehensive and transparent evaluation of their work.

When addressing Item XIII, consider the following points:

1. Study strengths: Identify and describe the key strengths of the study. These could include aspects such as a well-defined research question, rigorous study design, appropriate sample size, use of validated outcome measures, comprehensive data collection, or robust statistical analysis. Discuss how these strengths contribute to the reliability and validity of the study's findings.
2. Study limitations: Identify and describe the limitations of the study. These may include factors that could have influenced the results or introduced bias. For example, limitations could arise from the study design, data collection methods, participant recruitment or retention, measurement tools, or potential conflicts of interest. Clearly state the limitations and their potential impact on the study's outcomes.
3. Sources of bias: Identify and discuss potential sources of bias in the study. Common types of bias include selection bias, where the characteristics of the participants may not be representative of the target population; performance bias, where knowledge of the application may influence participant behavior; detection bias, where differences in outcome assessment occur between groups; and expectation bias, where preconceived beliefs or expectations influence participant and researcher behavior. Explain how these biases could have influenced the study's results and provide a rationale for their identification.
4. Mitigation of bias: If applicable, discuss any measures taken to mitigate potential sources of bias. This could include randomization and blinding procedures, standardized protocols for data collection and outcome assessment, or sensitivity analyses to assess the robustness of the results. Highlight any strategies employed to minimize bias and their effectiveness in addressing potential limitations.

By discussing the strengths and limitations of the study and identifying potential sources of bias, researchers can provide a comprehensive assessment of their work. This transparency allows readers to evaluate the reliability and generalizability of the study's findings while recognizing the potential impact of biases. A thorough discussion of study strengths and limitations contributes to the overall credibility and robustness of the research.

### Discussion and Conclusion - Item 16 (Conclusion)

*Provide a conclusion that accurately interprets study findings, including future perspectives.*

– Explanation & Elaboration –

Item 16 focuses on providing a conclusion that accurately interprets the findings of the study and offers insights into future perspectives. This item highlights the importance of aligning the conclusion with the main study objective and the primary and secondary outcomes. By summarizing the key findings and considering their implications, researchers can provide a concise and meaningful conclusion that contributes to the broader understanding of the study's implications and potential avenues for future research.

When addressing Item 16, consider the following points:

1. Summarize key findings: Provide a concise summary of the study's key findings, emphasizing the results related to the primary and secondary outcomes. Highlight the main conclusions drawn from the data analysis and present them in a clear and organized manner. Ensure that the conclusion aligns with the research question and objectives of the study.
2. Interpretation of findings: Offer an interpretation of the study findings, considering their implications within the context of the research field.
3. Future perspectives: Concisely state potential future directions or implications of the study findings. Consider how the results contribute to the existing knowledge base and identify gaps or areas that require further investigation. Highlight any recommendations for future research, clinical practice, or policy development.
4. Alignment with objectives and outcomes: Ensure that the conclusion accurately reflects the main study objective and the primary and secondary outcomes. Avoid overgeneralization or extrapolation beyond the scope of the study. Maintain focus on the specific research question and outcomes addressed in the study.

By providing a conclusion that accurately interprets the study findings and offers future perspectives, researchers can effectively communicate the significance of their work. This allows readers to understand the implications of the study and fosters the advancement of knowledge in the field.

### Statements - Item XIV (Funding and Conflicts of Interest)

*Disclose any potential conflict of interest, real or apparent, including the funding sources and their roles in the design, conduct, analysis and report of the study, potential role(s) of commercial companies, and personal conflicts of interest for each author.*

– Explanation & Elaboration –

Item XIV focuses on the disclosure of potential conflicts of interest associated with the study. This item emphasizes the importance of transparency and allows readers to evaluate any potential biases that may arise due to financial or personal relationships. It includes the disclosure of funding sources, their involvement in different stages of the study, the potential role(s) of commercial companies, and personal conflicts of interest for each author.

When addressing Item XIV, consider the following points:

1. Funding sources: Disclose all funding sources that supported the study. This includes grants, sponsorships, or financial contributions from governmental agencies, foundations, industry sponsors, or other sources. Specify the role of each funding source in the design, conduct, analysis, and reporting of the study. This information helps readers assess any potential influence of the funding sources on the study outcomes.
2. Involvement of funding sources: Describe the specific roles played by the funding sources in the study. This may include their involvement in the study design, data collection, data analysis, interpretation of results, and preparation of the manuscript. Provide details about any contractual agreements or relationships between the funding sources and the researchers.
3. Potential role(s) of commercial companies: Identify and disclose any potential role(s) of commercial companies, such as manufacturers of the XR application or related technologies, in the study. This may include provision of equipment, technical support, or other contributions. Clarify the extent of involvement and any potential conflicts of interest arising from these relationships.
4. Personal conflicts of interest: Each author should disclose any personal conflicts of interest that may influence the study. This may include financial relationships, consultancies, ownership interests, or other relevant affiliations with organizations or entities that could be perceived as influencing the study outcomes. Authors should provide comprehensive information regarding their own potential conflicts of interest.

By disclosing potential conflicts of interest, authors demonstrate transparency and enable readers to assess any potential biases or influences on the study findings. This information is crucial for evaluating the objectivity and credibility of the research and ensuring public trust in the study outcomes.

### Statements - Item 17 (Application)

*Indicate whether the application is a commercial product, it is publicly available, it can be accessed, it complies with the medical device regulations and whether the application was approved for its intended use by a formal regulatory body or if the study is part of the clinical evaluation for future certification.*

– Explanation & Elaboration –

Item 17 focuses on providing information about the commercial availability, accessibility, regulatory compliance, and approval status of the XR application being studied. This item aims to inform readers about the application's status as a commercial product, its availability to the public, compliance with medical device regulations, and whether it has received approval from a formal regulatory body for its intended use. By addressing this item, researchers provide important contextual information about the application and its regulatory status.

When addressing Item 17, consider the following points:

1. Commercial product status: Indicate whether the XR application being studied is a commercial product, i.e., whether it is developed and marketed by a commercial entity for use in healthcare or other related domains. Specify the name of the application and, if applicable, the company or organization responsible for its development.
2. Public availability and accessibility: Describe whether the application, or the source code, is publicly available and accessible. Provide information about how individuals can access and use the application, such as through online platforms, app stores, or specific healthcare settings.
3. Compliance with medical device regulations: Discuss whether the XR application complies with relevant medical device regulations, standards, and guidelines. These regulations may vary depending on the country or region where the study was conducted. Highlight the importance of complying with these regulations to ensure the safety, efficacy, and quality of the application.
4. Regulatory approval: Specify whether the XR application has received approval for its intended use from a formal regulatory body. This may include clearance from regulatory agencies, such as the U.S. Food and Drug Administration (FDA) or the European Medicines Agency (EMA), or adherence to specific medical device directives or regulations in other jurisdictions.
5. Clinical evaluation for future certification: If the study is part of a clinical evaluation aimed at future certification or regulatory approval, provide details about the regulatory pathway and the stage of the evaluation process. Explain whether the study is intended to gather evidence for future submission to regulatory authorities.

By providing information about the commercial availability, accessibility, regulatory compliance, and approval status of the XR application, researchers offer valuable insights into the application's context and regulatory oversight. This information helps readers understand the broader landscape in which the study was conducted and the implications of the application's regulatory status on the study's findings and future use.
